# PSFC: a Pathway Signal Flow Calculator App for Cytoscape

**DOI:** 10.12688/f1000research.6706.2

**Published:** 2017-04-03

**Authors:** Lilit Nersisyan, Graham Johnson, Megan Riel-Mehan, Alexander R Pico, Arsen Arakelyan

**Affiliations:** 1 Institute of Molecular Biology NAS RA, Yerevan, Armenia; 2 California Institute for Quantitative Biosciences (QB3), San Francisco, CA, 94158, USA; 3 University of California, San Francisco, CA, 94158, USA; 4 Gladstone Institutes, San Francisco, CA, 94158, USA; 5 American University of Armenia, Yerevan, Armenia

**Keywords:** pathway signal flow, cytoscape, systems biology, networks, scoring algorithms, gene expression

## Abstract

Cell signaling pathways are sequences of biochemical reactions that propagate an input signal, such as a hormone binding to a cell-surface receptor, into the cell to trigger a reactive process. Assessment of pathway activities is crucial for determining which pathways play roles in disease versus normal conditions. To date various pathway flow/perturbation assessment tools are available, however they are constrained to specific algorithms and specific data types. There are no accepted standards for evaluation of pathway activities or simulation of flow propagation events in pathways, and the results of different software are difficult to compare. Here we present Pathway Signal Flow Calculator (PSFC), a Cytoscape app for calculation of a pathway signal flow based on the pathway topology and node input data. The app provides a rich framework for customization of different signal flow algorithms to allow users to apply various approaches within a single computational framework.

## Introduction

Cell signaling pathways are sets of directed interactions between biological molecules, that are initiated by a particular signal (e.g. a ligand binding to a receptor) and result in realization of certain target processes (e.g. transcription of genes). Pathways can be represented as graphs, with nodes as biological entities (proteins, other biomolecules, chemical compounds, other pathways), and edges as physical or regulatory interactions between them. In contrast to protein-protein interaction networks, biomolecular pathways have directionality, input nodes, intermediate nodes and branches, and output or sink nodes.

*Pathway Signal Flow (PSF)*, *or perturbation*, is the flux generated by propagation of the signal starting from input nodes, flowing through intermediate nodes in branches and accumulating at sink nodes. Thus, PSF can be an indicator of pathway activity state. Assessment of changes in pathway activity is of major interest for identification of processes involved in the formation of certain phenotypes (healthy and diseased states), and assessment of cell response to drugs and other stimuli. First attempts to globally evaluate the pathway activity changes were performed in parallel with the appearance of high-throughput gene expression measurement experiments. Pathway involvement is typically analyzed by over-representation analysis (ORA) ^1^ or gene set enrichment analysis (GSEA) ^2^. The major drawback of these widely used approaches is that they operate on gene sets involved in the pathway, but do not account for the pathway topology and ignore the interactions between the nodes.

A number of techniques and tools have recently emerged, aimed at determining pathway activities based on topological information of pathways and gene expression/protein activity levels. One of the pioneering papers in this direction was the Pathway Impact Analysis algorithm, which combines GSEA with gene position in the network ^3^. Other approaches apply specific rules to model flow or signal propagation through the pathway and evaluate the amount of the signal reaching the sink nodes ^4– 6^.

The above mentioned algorithms and tools are implemented using various programming and scripting languages, making their use and result comparison difficult in the common context. Moreover, they often work with programming environment specific objects, and are not flexible for using biological pathways that appear in various formats. Cytoscape, on the other hand, is a powerful and flexible platform that, together with its diverse collection of available apps, provides a rich environment for parsing, visualization and analysis of networks ^7^.

Herein we present Pathway Signal Flow Calculator (PSFC), a Cytoscape app for computation of pathway signal flow based on input data and pathway topology. PSFC provides a variety of options for signal propagation, both used in already published signal flow algorithms ^3– 6^, as well as in new ones. Thus, it allows experimenting with the results obtained by various (existing and customizable) approaches within a single framework, and evaluating their ability to simulate real life situations.

## Methods

### Implementation

**Figure 1.**
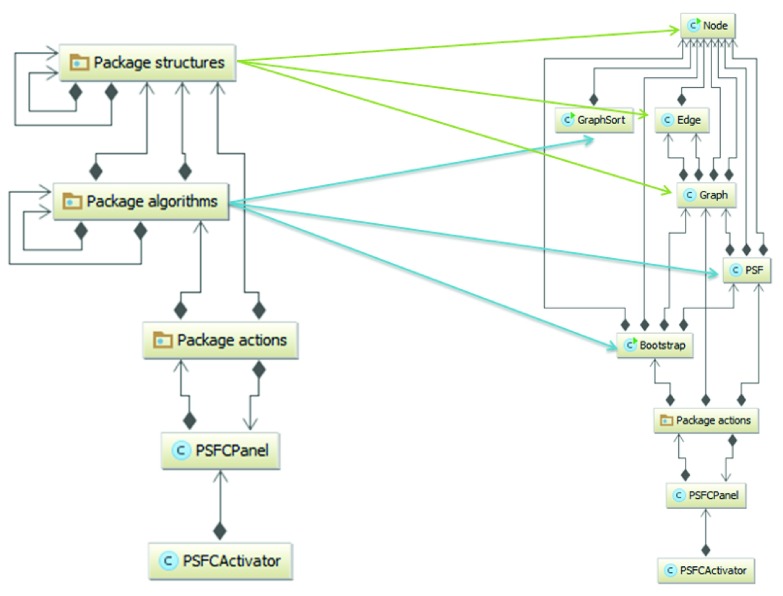
The PSFC class diagram. Only the main packages are displayed. In the right-hand diagram, the packages *logic.algorithms* and *logic.structures* are extended to respective classes. The communication from and to the user is performed via the *PSFCPanel* class and the *gui.actions* package. PSFC inherent structures and algorithms are implemented in the packages *logic.algorithms* and *logic.structures*. The classes extended from the packages *logic.structures* and *logic.algorithms* are shown with green and blue arrows, respectively. Class dependencies are indicated with grey arrows.

***PSFC packages and data structures***. PSFC is implemented in Java and is available as an app for Cytoscape 3. The main module consists of two main packages, *logic* and *gui*. The package *logic* is designed to handle PSFC-inherent structures and algorithms, while the *gui* package is responsible for user communication via the PSFC tab in the Cytoscape GUI west panel, and for mapping Cytoscape inherent data structures to PSFC data structures ( *Graph, Node* and *Edge*) contained in the *logic* package ( Figure 1).

### PSFC algorithms

***Graph sorting***. Graph sorting is the first step before proceeding to signal flow calculation. The aim of sorting is to assign levels to the nodes, to propagate the signal from lower to higher level nodes.

We have modified the topological sort algorithm implemented in Java JGraphT library [ http://jgrapht.org/], to handle multiple input node containing graphs.

Recall that biological networks often contain feedback loops, which create cycles in graphs. PSFC firstly performs depth first search traversal and removes backward edges from the graph, and performs the topological sorting on the resulting acyclic graph, after which the backward edges are restored. Finally, node levels of the sorted graph are mapped to the Cytoscape node attributes table.

**Figure 2.**
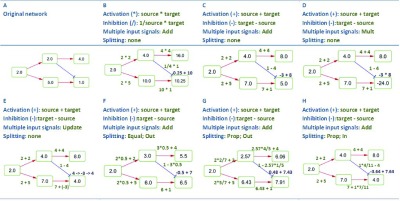
Demonstration of PSF computation on a sample network with different flow propagation rules. Red and blue edges are of types “activation” and “inhibition”, respectively. Multiple signals at a target node are computed by addition (Add), multiplication (Mult), or by updating target node signals (Update). Signal splitting is either set to “none” or “equal” (Equal) or “proportional” (Prop) rules, and is either performed on multiple outgoing edges (Out) or multiple incoming edges (In).

***Pathway signal flow calculation***. In biological signaling networks, the signal is propagated via interactions between source-target node pairs. The outcome of signal propagation events is the signal (PSF value) accumulated at each network node. Figure 2 provides an example of how the signal propagates through a sample network, with various signal propagation options applied.

***Rules for simple source-target interactions***. Functional interaction types can be broadly defined as activation or inhibition, while the range of physical and regulatory interactions is much wider (phosphorylation, binding, dimerization, ubiquitination, etc.). An edge in a graph carries a signal transfer function, which depends on the interaction type. PSFC allows the user to define the interaction type of each edge in the network, as well as define the edge-type specific mathematical functions of wide complexity. These functions should have the form *f* ( *source*, *target*), where *the source* and *target* variables stand for the source node signal and the target node value. The functions are parsed with Exp4j Java library for symbolic operations [ http://www.objecthunter.net/exp4j/]. Function assignment for different edge types is shown in Figure 2.

***Rules for multiple incoming and outgoing signals***. Generally, the intensity of interactions between molecules largely depends on their concentration and activation state. However, if a node has several interacting partners, those may compete with each other, and the interaction capacity of the node may be “split” between those partners. Thus, there is the option to proportionally split the signal among multiple edges starting from a single source or ending on a single target node. The signals on multiple edges ending on a single target node may be processed in one of the following three ways: the signals may be computed separately at each edge and added (i) or multiplied (ii) to each other, or they may be processed in order (iii), by updating the signal at a target node each time a single edge is processed. The order, in which the edges are processed in the last case, may be adjusted by user defined edge ranks ( Figure 2). The example network presented in Figure 2, and instructions for replicating the rules and results are provided in the Supplementary material, figure2_inputs_and_instructions.rar.

**Figure 3.**
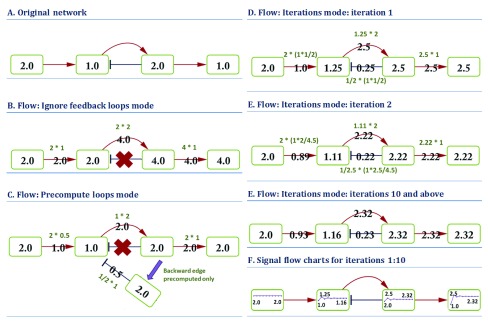
Signal flow calculation on a sample network with different options for loop handling. The PSF flow rules are: for red edges of type activation (*): source * target; for blue edges of type inhibition (/): 1/source * target; Multiple input signals: *Addition*; Splitting: *Proportional*; Split on: *Incoming edges*. The “Ignore feedback loops” mode ( **B**) does not account for backward edges. In the “Precompute loops” mode ( **C**), the backward edge signal first updates the target node value, and is ignored in the following single iteration. ( **D**– **E**): signal flow at different iterations with “Iterate until convergence” mode. The network converges at iteration 10. The dynamics of flow changes at each node during 10 iterations are shown as line charts in ( **F**). Edges ignored during the computation are indicated by red *X* symbols.

***Handling of feedback loops***. The presence of negative and positive feedback loops in biomolecular pathways is of paramount importance for pathway functionality and regulation. However, currently it is a major obstacle for developing optimal algorithms for pathway activity assessment. To our knowledge, there is no single solution for treatment of loops in signal propagation algorithms, thus PSFC provides several options for loop handling:

*Ignore feedback loops:* In this case cycle-forming backward edges are ignored during PSF calculations ( Figure 3B).

*Precompute signals at loops:* In this mode, the algorithm firstly finds cycle-forming backward edges, computes their signals, and updates their target node values. Afterwards, the algorithm runs on the whole graph in the “ignore feedback loops” mode ( Figure 3C).

*Iterate until convergence:* The algorithm runs for several rounds, until convergence of signal flow values is reached ( Figure 3D–F). Convergence is reached if the percentage of signal changes between two iterations is less than the specified convergence threshold at all the nodes. If convergence is not achieved, the algorithm stops after running for a defined number of iterations. The user may check the convergence status of the calculations in the PSFC log file and in the command prompt.

The example network for replicating the loop handling options presented in Figure 3 is available in the Supplementary material, loop_example.xml.

***Significance calculation***. The significance value of signal flows at each node is computed using bootstrapping. The user may choose between sample-centric or gene-centric bootstrapping modes. In the sample-centric mode, the values of the nodes in the network will be reshuffled among each other during resampling. In the gene-centric mode, the value of each node is randomly chosen from a supplied distribution of node values, e.g., from measurements of a given gene’s expression across multiple samples.

### Operation

PSFC is implemented for Cytoscape version 3.2 and higher, with Java 1.7 or higher. PSFC may be installed with either the Cytoscape App Manager or by direct download of the jar file from http://apps.cytoscape.org/apps/psfc. The whole functionality of PSFC is accessible to users via a single tab in the Cytoscape GUI west panel.

### Use cases

**Figure 4.**
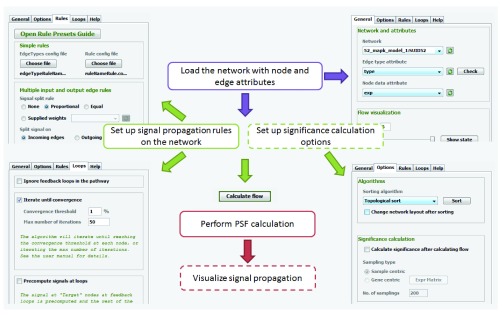
The general use case of PSFC. The user should load the network into Cytoscape, and import node and edge attributes using the Cytoscape environment. Further, the user sets the rules for signal propagation, loop handling and significance calculation. After PSF calculation, the signal propagation may be visualized in Cytoscape. The dashed rectangles are optional.

***General use case of PSFC***. The main use case of the app is presented in Figure 4. PSFC operates on any network loaded into the Cytoscape environment. Node data and edge types should be loaded into Cytoscape attribute tables, while signal propagation rules should be set in respective PSFC GUI tabs ( Figure 4). PSF computation is performed with the “Compute flow” button. The resulting PSF values are stored both in Cytoscape attribute tables, and in PSFC output files (the score backup file and psfc.log file in text formats). The signal propagation may be visualized via node color and edge width mapping, where continuous values are mapped to color gradients and width ranges at a chosen level or across all levels in a sequence.

***PSF calculation on MAPK signaling pathway: a use case***. We evaluated signal flow changes in the MAPK signaling pathway network taken from previously published papers ^8, 9^. In their paper, Nelander *et al.* have performed a series of experiments, where they have downregulated one or many of the MAPK pathway proteins, and measured the changes of protein phosphorylation levels, and the states of G1-arrest and apoptosis ^8^. Feiglin *et al.* ^9^ have compared the results of the experimental data with their predictions, based on a wiring algorithm described in their paper ^9^. We have repeated the same experimental simulations, to compare the performance of PSFC with the wiring algorithm and with the experimental data. The node values were presented as gene expression fold change (FC) values that show the relative increase or decrease of gene expression compared to the reference state. In the reference state, the amount of PSF should be 1, corresponding to the normal level of pathway activity necessary to realize the target biological process. Departure of PSF values from 1 is indicating an up- or down-regulation of the pathway. To simulate this situation, we have applied the following rules for signal propagation. The single edges were treated with (source*target) and (1/source*target) functions for edges of types activation and inhibition, respectively, ensuring that an FC change on a node propagates proportionally via signal perturbations to downstream nodes. Furthermore, we have applied splitting on incoming edges and addition of multiple incoming signals on a single target node. This is based on the speculation that, in signaling pathways, the capacity of a protein to interact with upstream agents depends on the relative frequency of co-occurrence and the interaction strength with those agents, which is this case, is represented as the PSF signals of the source nodes. Finally, loop handling was in “iterate until convergence” mode, since the absence of positive feedback loops in the MAPK pathway and FC representation of the node values ensure that the algorithm will converge.

**Figure 5.**
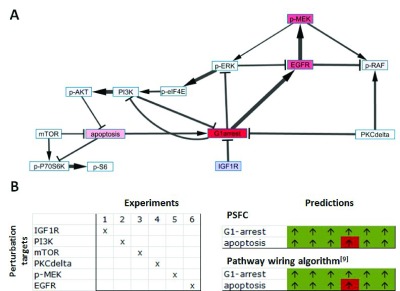
PSF calculation results on MAPK signaling pathway. MAPK signaling pathway is generated by the model used in 8. **Part A** shows the pathway, as visualized in Cytoscape after PSF calculations. Edges of activation and inhibition types have Delta and T shaped target arrows. The darker colors of nodes indicate greater PSF values, and the line width corresponds to the signal intensities at the edges. The prediction results of six perturbation experiments by PSFC and the pathway wiring algorithm ^9^ are presented in **part B**, similar to the representation in 9. In each of the six experiments, one node was down-regulated, as indicated by the “x” sign. The predicted perturbations at the “G1-arrest” and “apoptosis” nodes are shown with up and down arrows. Green and red colors indicate consistency and non-consistency, respectively, of the predictions with the experimental results presented in 8.

We have performed PSF calculations in 6 different experiments. In each of these experiments one of the IGF1R, PI3K, mTOR, PKCdelta, p-MEK, or EGFR nodes was assigned a value of 0.1 (down-regulated), while the rest of the nodes had “fc” values of 1, which corresponds to the unchanged state compared to the control ( Figure 5A).

Under all the experimental settings we have predicted up-regulation of the “G1-arrest” and “apoptosis” nodes, which is in full accordance with the predictions of Feiglin *et al.* ^9^. These predictions deviated from the experimental outcomes ^8^ in only one case ( Figure 5B). The network xml file and all configuration files are available in Supplementary material, MAPK_psfc_configurations.rar.

## Summary

We have developed PSFC, a Cytoscape app for PSF calculation. The main purpose of the app is to evaluate the signal flow propagation in pathways and assess activity states of pathway components, based on input data and the topology. PSFC may be used for the purpose of assessment of pathway activity deregulations in different conditions, for simulation studies on network dynamics, etc.

Compared to other similar software, PSFC stands out with a wide set of rules and options for signal propagation, which makes it possible to use the app for the majority algorithms that could possibly be applied for pathway flow calculations in different biological contexts. It is, thus, not constrained with preset algorithm design, but allows users to apply their own algorithms. Thus, PSCF can be used in routine data analysis by bench biologists using available presets, but also can become a powerful tool for sophisticated pathway analyses in the hands of a bioinformatics skilled person.

## Software availability

Software available from: http://apps.cytoscape.org/apps/psfc

### Latest source code

https://github.com/lilit-nersisyan/psfc/

### Source code as at the time of publication

https://github.com/F1000Research/PSFC

Archived source code as at the time of publication [version1 of manuscript]

http://dx.doi.org/10.5281/zenodo.19465 ^10^

Archived source code as at the time of publication [version 2 of manuscript]

https://doi.org/10.5281/zenodo.439419 ^11^

### License

PSFC is free software; it can be distributed and/or modified under the terms of the GNU General Public License version 3. The license can be found at http://www.gnu.org/licenses/gpl.html. The exp4j library is distributed under Apache License 2.0, while the JGraphT library is dual-licensed GNU Lesser General Public License and Eclipse Public License.

## Supplementary material

### Archive for 'PSF calculation on MAPK signaling pathway: a use case'

The archive contains source and configuration files used to generate the data described in the “PSF calculation on MAPK signaling pathway: a use case” subsection of the manuscript. The MAPK_network.xml file contains the network model, which should be imported into Cytoscape. The EdgeTypeRuleName.config and RuleNameRule.config files are configuration files defining simple rules for flow propagation. The psfc.props contains the properties used by PSFC during calculations. The Flow_propagation_rules.pdf describes the applied rules and options.

### PSFC user manual

The user manual covers installation notes, a generic use case and a specific example, in addition to an overview of the graphical user interface and details about network sorting, flow rule configurations, pathway flow calculation and flow visualization. This supplemental file is a static version of the manual. For the latest online version, click on the Tutorial link on the PSFC app page: http://apps.cytoscape.org/apps/psfc.

**Figure2_inputs_and_instructions.rar**

Network file and instructions to replicate the examples of Figure 2.

The archive contains the following files:

InstructionsToFigure2.pdf: contains instructions to load the files in the archive and replicate the examples in Figure 2.

figure2.xgmml: the network file that can be imported into Cytoscape.

figure2.rules a property file containing the proper settings to replicate the examples of Figure 2

edgeTypeRuleName1.config, edgeTypeRuleName2.config, ruleNameRule – standard configuration files for PSFC

Loop example xml

A toy network to replicate loop handling options presented in Figure 3.

The xml file can be imported into Cytoscape. The loop handling options can then be applied as described in the manuscript and the legends of Figure 3.
