# Supplementary material for: PSFC: a Pathway Signal Flow Calculator App for Cytoscape
Source: F1000Res. 2017 Apr 3;4:480. Originally published 2015 Aug 5. [Version 2] doi: 10.12688/f1000research.6706.2 (PMC4706054; doi:10.12688/f1000research.6706.2)
Supplement: Supplementary file 2 [file f1000research-4-12210-s0003.tgz › 14137802-240d-47fd-aba5-95e92e3aafd2.pdf]

2SFC 1.1.2 USER MANUAL

Lilit Nersisyan and Arsen Arakelyan,

Institute of Molecular Biology NAS, Armenia

Graham Johnson, Megan Riel-Mehan, Alexander Pico

UCSF and Gladstone Institutes, USA

March, 2017

PSFC User Manual

Table of Contents

PSFC User Manual 1

Introduction 3

Development 3

Citation 3

License 3

Release 1.1.2 4

Getting started 5

Installation 5

General use case 6

1. Load the network 6
2. Load node input data 6
3. Load edge types attribute 7
4. Define flow propagation rules 7
5. Define loop handling options 7
6. Significance calculation options 7
7. Flow calculation 7
8. Signal flow visualization 7
9. Final output of PSFC 7

Getting started example: the MAPK signaling pathway 9

1. Load the network 9
2. Load node and edge attributes. 10
3. Set signal propagation rules 11
4. Set loop handling options 12

5. Calculate flow 13

6. PSFC output 14

7. Visualize the flow 15

GUI components 17

General tab 17

Options tab 20

Rules tab 22

PSFC algorithms 26

Network sorting 26

Flow propagation rules 27

Pathway flow calculation 29

Flow visualization 29

Introduction

The Pathway Signal Flow Calculator (PSFC) is a Cytoscape app for calculation of pathway signal flow based on input data and pathway topology. The app provides a flexible interface for setting flow propagation rules and applying algorithms of wide range to assess activity states of pathway nodes (PSF signals). The app supports visualization of signal propagation on the network.

Development

The app has been developed by Lilit Nersisyan and Arsen Arakelyan, from the Bioinformatics Group at the Institute of Molecular Biology of the National Academy of Sciences of the Republic of Armenia (IMB NAS RA) in collaboration with Graham Johnson, and Megan Riel-Mehan from University of California, San Francisco, and Alexander Pico from Gladstone Institutes.

The project was funded by Google Summer of Code 2014, and Armenian National Science and Educational Foundation Grant research grant molbio-3818 (PI: LN).

Citation

When using the app in your research, please link to the app's webpage at
http://apps.cytoscape.org/apps/psfc and cite our paper as:

> Nersisyan L, Johnson G, Riel-Mehan M et al. PSFC: a Pathway Signal Flow Calculator App for Cytoscape [version 1; referees: 1 approved] F1000Research 2015, 4:480 (doi: 10.12688/f1000research.6706.1)

License

Copyright © 2017

Lilit Nersisyan, IMB NAS RA

Arsen Arakelyan, IMB NAS RA

Graham Johnson, UCSF

Megan Riel-Mehan, UCSF

Alexander Pico, Gladstone Institutes

This program is free software: you can redistribute it and/or modify it under the terms of the GNU General Public License version 3. The license can be found at http://www.gnu.org/licenses/gpl.html .

Release 1.1.2

What's new in 1.1.2 (compared to 1.0.2):

Major points:

Run PSF for multiple columns

In 1.1.2, the user is able to simultaneously select multiple columns containing node data from multiple samples to run PSF on all of them in succession, as opposed to performing many PSF computations manually.

Operator nodes

In version 1.1.2, the user may specify signal transfer functions not only on edges, but also on specific nodes. In this way, each node may appear as in 'operator' that takes input signals from multiple incoming edges and processes them in a unique way. This is particularly useful for:

a) Protein complexes: usually the presence of all the complex subunits is mandatory for it to function. In this case the user may set the 'min' function onto the complex node. This means that the subunit with the minimum abundance will be the limiting factor for the activity of the whole complex.
b) Many proteins/complexes compensating each other's functions. In situations when the presence of either protein/complex is enough for the signal to transduce, the user may create an 'operator' node and assign the 'max' function to it.
c) Sometimes, the 'mean' (average signal) and the 'prod' (product of signal multiplications) is desirable, if the user is interested in general state of the summary states of input signals.

Minor points:

- PSF visualization is now fully customizable, with the option to set the minimum and maximum SPFs to the desired value.
- Many bug fixes.

Getting started

PSFC has been tested to work with Cytoscape version 3.2.0 and higher on 64 bit systems. Running it on other systems or with previous versions of Cytoscape may result in errors.

Installation

In Cytoscape, go to Apps -> "App Manager", choose PSFC under and click on the install button. In case of successful installation, the "PSFC" app tab should appear in the west panel of Cytoscape (Figure 1).

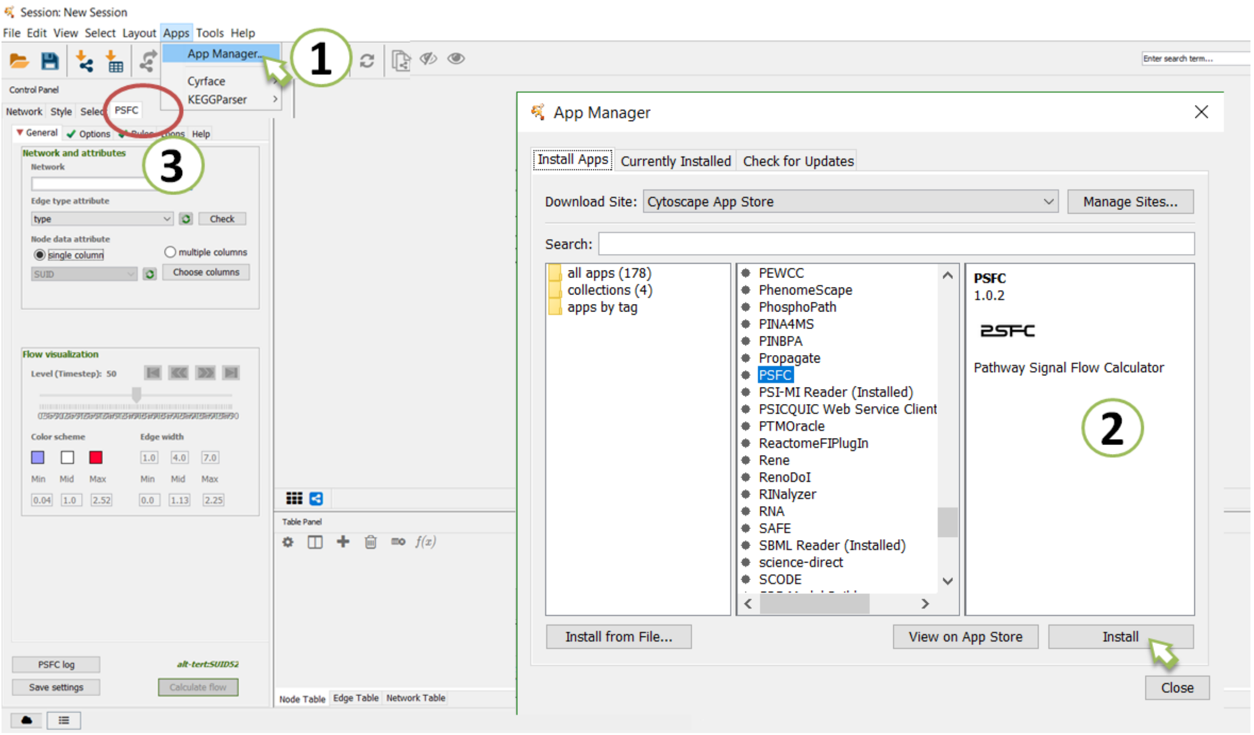

Figure 1. Installation of PSFC.

General use case

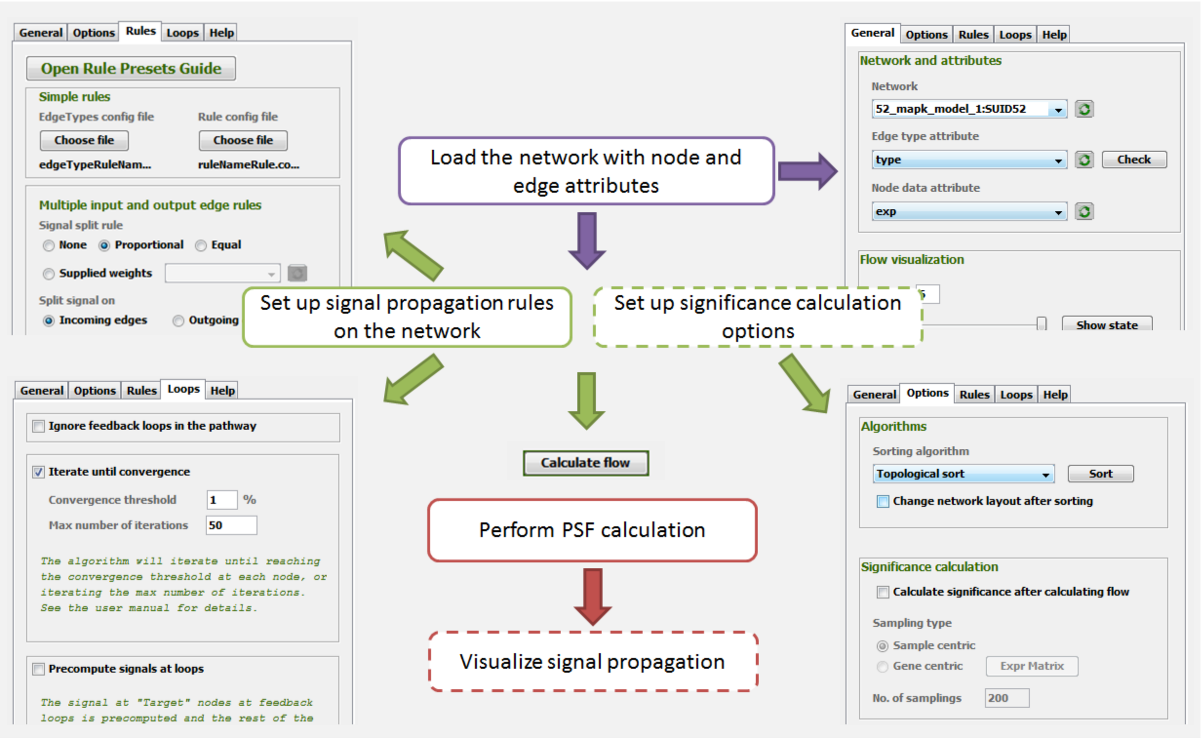

Figure 2. The general use case of PSFC.

1. Load the network

PSFC works on any pathway network loaded into Cytoscape environment. The network may either be loaded with File -> Import -> Network from a local file, or from the web, with available pathway parser apps, such as WikiPathways, CyKEGGParser, KEGGScape, ReactomeFIPlugin, etc. Instructions on how to load, create and edit networks can be found at Cytoscape User Manual page: http://wiki.cytoscape.org/Cytoscape_3/UserManual#Cytoscape_3.2BAC8-UserManual.2BAC8-Quick_Tour.Welcome_Screen.

2. Load node input data

The nature of node data may vary: it can be absolute or relative expression of genes in each node, fold change of expression values, node ranks, protein concentration, protein phosphorylation level, etc. Basically, this is any data related to the activity states or the quantities of the nodes. The node data should be loaded in Cytoscape node attribute table. This can be done manually in Cytoscape Table Panel , via File -> Import -> Table from a local file. For more details on how to work with node attributes, the user is referred to Cytoscape User Manual page: http://wiki.cytoscape.org/Cytoscape_3/UserManual#Node_and_Edge_Column_Data.

3. Load edge types attribute

The edge types actually define how the signal is propagated between nodes in the network. The type of the edge should be loaded in Cytoscape edge attribute table, similar to node attributes, as described above. As explained below, each edge type may be associated with a different mathematical function.

4. Define flow propagation rules

After step 2-3, each node is assigned a value, and each edge is described with a specific behavior. Flow propagation rules define: (i) how the signal is passed from a source node to a target node via an edge; and (ii) how nodes receiving multiple signals from multiple source nodes process those signals, and vice-versa. These rules are defined upon the user's choice.

5. Define loop handling options

Biological pathways contain positive and negative feedback loops. In PSFC provides a number of options for handling loops in pathways.

6. Significance calculation options

The output of signal flow calculation is PSF values assigned to each node that describes the activity state of the node. The significance of the PSF values is calculated with Bootstrap resampling. The user may choose to compute significance values, and may select different options for resamplings.

7. Flow calculation

After setting the configurations, PSF computation is performed with a click of the "Compute flow" button. PSFC outputs PSF values for all the nodes and edges in the pathway. These values describe the amount of signal that a node carries and an edge transfers.

8. Signal flow visualization

The signal propagation may be visualized on the network via node color mapping and edge width mapping, according to node and edge signal values.

9. Final output of PSFC

The PSF values of nodes and signals at edges are mapped to Cytoscape node and edge attribute tables, and are also written in a backup file.

The following columns appear in the Node attribute table:

- *psfc.level* .

This column appears after network sorting. The level of each node is equal to the distance of that node to the pathway input. The input nodes have level 0, the sink nodes have the highest levels in their branch.

- *psf_l(x)*

  These columns are used for signal flow visualization, and may be of little interest to the user. The signal flow propagation is performed from 0 level nodes to higher level nodes. After a level *x* is processed, a column psf_l*x* appears, where the node PSF (signal) values are kept. For example, in psf_l2, only 0, 1 and 2 levels are processed, and the PSF (signal) values of nodes of those levels are updated, while the rest of the nodes have PSF values equal to their original input values. Note that when performing many iterations, only values of the last iteration are displayed.

- *psf_final*

  This column contains the final PSF values of all the nodes, when all the levels are processed. In case of many iterations of PSF calculations, the PSF values of the last iteration are displayed.

- *psfc_pval*

  The significance p values of the PSF values for each node.

The following columns appear in the Edge attribute table:

- *psfc_isbackward*

  This column appears after network sorting. If true, then the edge is a loop forming backward.

- *psfc _l(x)*

  These columns are used for signal flow visualization, and may be of little interest to the user. The signal flow propagation is performed from 0 level nodes to higher level nodes. After a level *x* is processed, a column psf_l*x* appears, where the edge signals in the current state are kept. For example, in psf_l2, only 0, 1 and 2 levels are processed, and the signals of the edges of those levels are updated, while the rest of the edges have signals equal to 1. Note that when performing many iterations, only values of the last iteration are displayed.

Getting started example: the MAPK signaling pathway

This is a quick demonstration of the app workflow. The MAPK signaling pathway structure is taken from previous publications:

1. Nelander S, Wang W, Nilsson B, *et al*: Models from experiments: combinatorial drug perturbations of cancer cells. *Mol Syst Biol.* 2008; **4**: 216.

2. Feiglin A, Hacohen A, Sarusi A, *et al*: Static network structure can be used to model the phenotypic effects of perturbations in regulatory networks. *Bioinformatics*. 2012; **28**(21): 2811-8.

To follow the demo, go to http://big.sci.am/apps/psfc/MAPK_psfc_configurations_v2.rar, and download the model network, configuration files, as well as the Excel output file, for comparison of results.

1. Load the network

Go to File -> Import -> Network -> File, and navigate to “MAPK_model.xml” directory, click Open to load the network into Cytoscape.

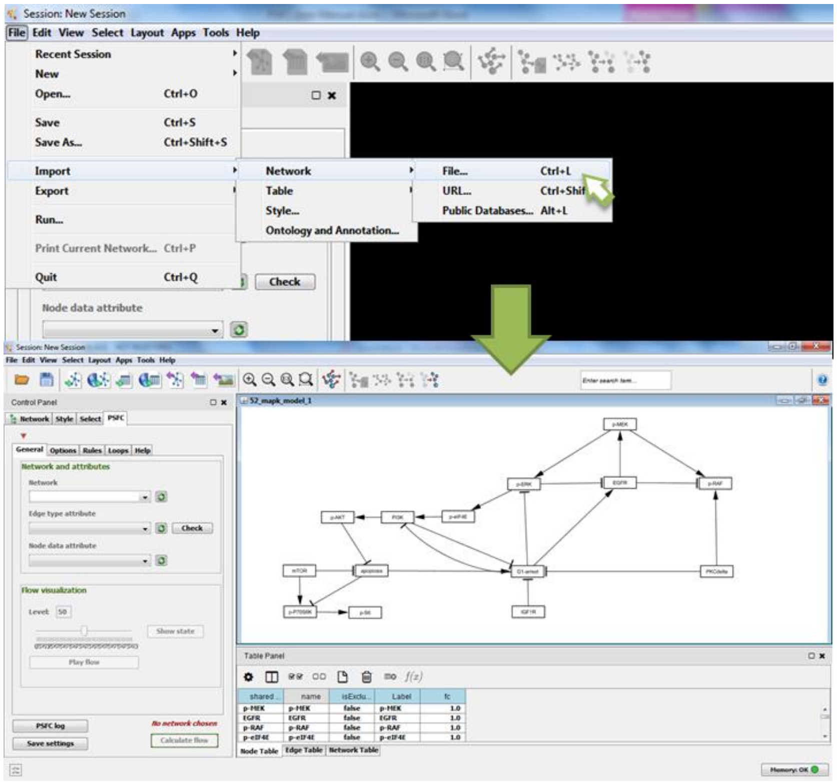

Press on the refresh buttons in the PSFC “General” tab, to notify it about the newly loaded network and attributes. From the **Network** dropdown menu choose the MAPK network.

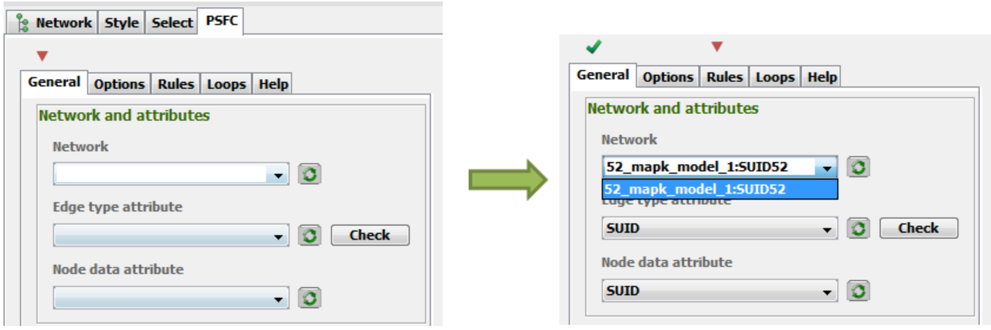

2. Load node and edge attributes.

The MAPK network contains two types of edges: activation and inhibition. In the following steps, each of these edge types will be assigned a different mathematical function for signal transfer. The edge type attribute is loaded in the Cytoscape edge attribute table, in the **type** column. In PSFC **General** tab, select the **type** column from the **Edge type attribute** drop-down menu.

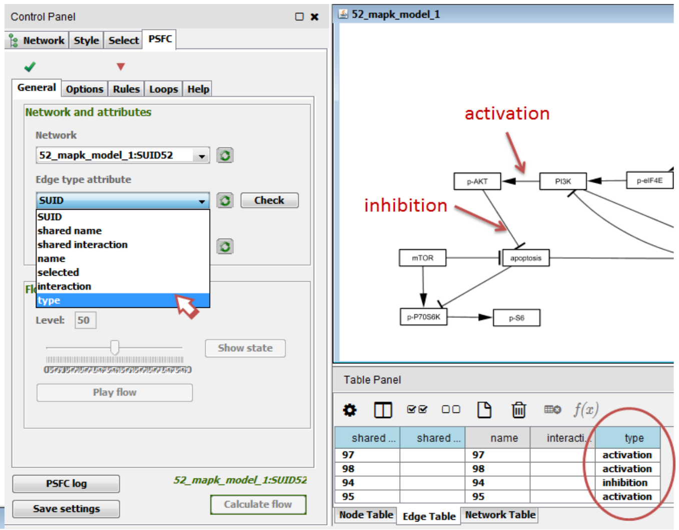

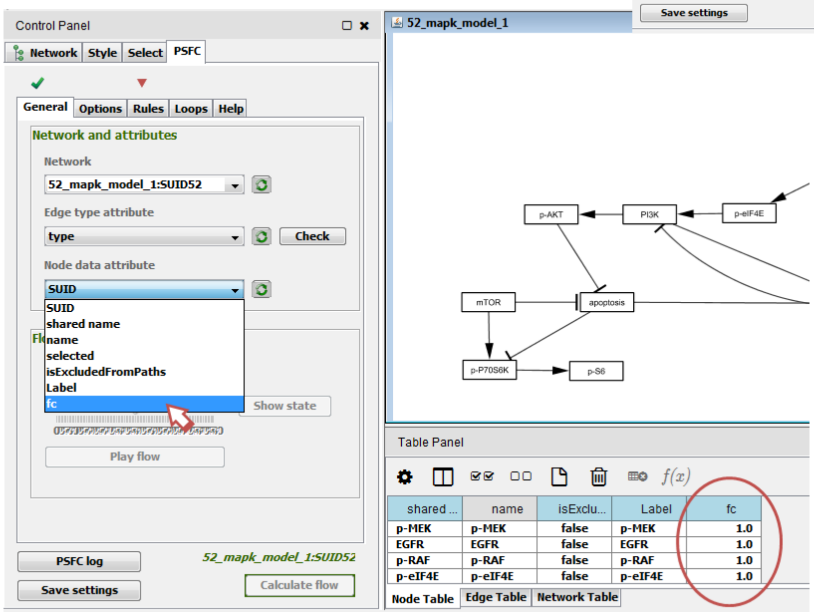

The node data is in the node attribute table, under the column **fc.** The values in this column represent fold changes of protein phosphorylation levels in a studied condition compared to the control.

In our network, all the nodes have initial values of 1.0 that correspond to the control (not changed) state. Furthermore, we will change the values of different nodes of the pathway, to see how these changes propagate to other nodes and to the sink nodes of the pathway. From the **Node data attribute** drop-down menu in the PSFC **General** tab, choose the **fc** column.

3. Set signal propagation rules

Simple rules

In biological pathway representations, an edge is a connector between a source and a target node, which transfers the signal from the source to the target. Edges may have different functional annotations. In our network, there are edges of type activation that increase the activity of the target node, and those of type inhibition, that decrease it. We would like to simulate these functional annotations of edges by defining a function for each of those. For this we will go the **Rules** tab **Simple rules** panel. There are two configuration files: the **Edge types config file** is a tab separated file, where each edge type is assigned a function name. The mathematical functions corresponding to each of these function names are defined in the **Rule config file.** Those are in the form *f(source, target),* where *source* and *target* variables are source signal and target value respectively.

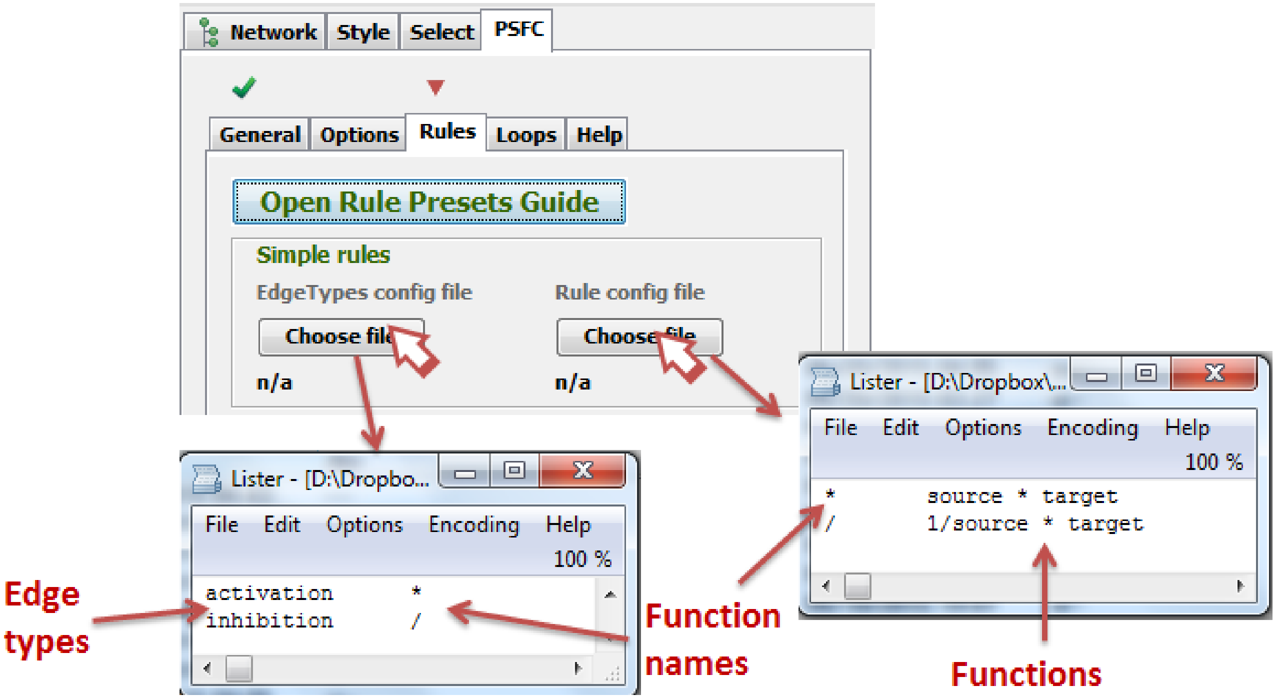

Multiple input and output edge rules

Following these simple rules for single edges, we should also define how the signal should be transferred if a single source node has multiple outgoing edges, or many edges are incoming to the same target node. These rules are set with the **Multiple input and output edge rules** panel in the **Rules** tab. For details on various rules in the panel, refer to further sections of the manual. We will now discuss the rules set for the MAPK signaling pathway.

- The **Split rule** is set to **Proportional** and the **Split signal on** is set to **Incoming.** This means that the signal coming from multiple edges to the same target node, will be split or assigned weights, based on relative values of their source nodes. Biologically, this simulates the fact that when interacting with multiple competing partners, the interaction intensity of the molecule depends on relative concentrations/activity states of those partners.
- The **Multiple signal processing rule** is set to **Addition.** With this rule, the signals from multiple edges coming to the same target node will be added to each other, and the sum will be the signal transferred to the target edge.

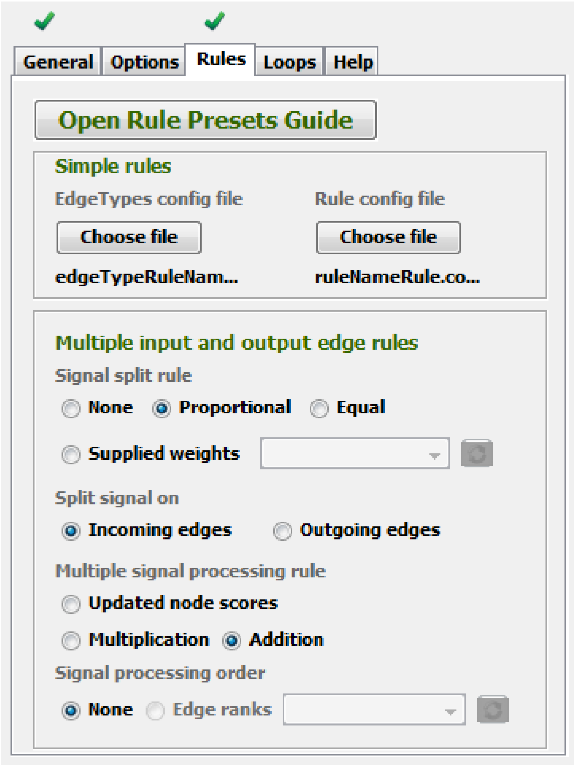

4. Set loop handling options

Biological pathways contain positive and negative feedback loops, which are important regulators of pathway activity and properties. The MAPK signaling pathway contains many negative feedback loops. Those can be seen by applying the Topological sorting algorithm by clicking the **Sort** button in the **Options** tab, and checking the **Change network layout after sorting** checkbox.

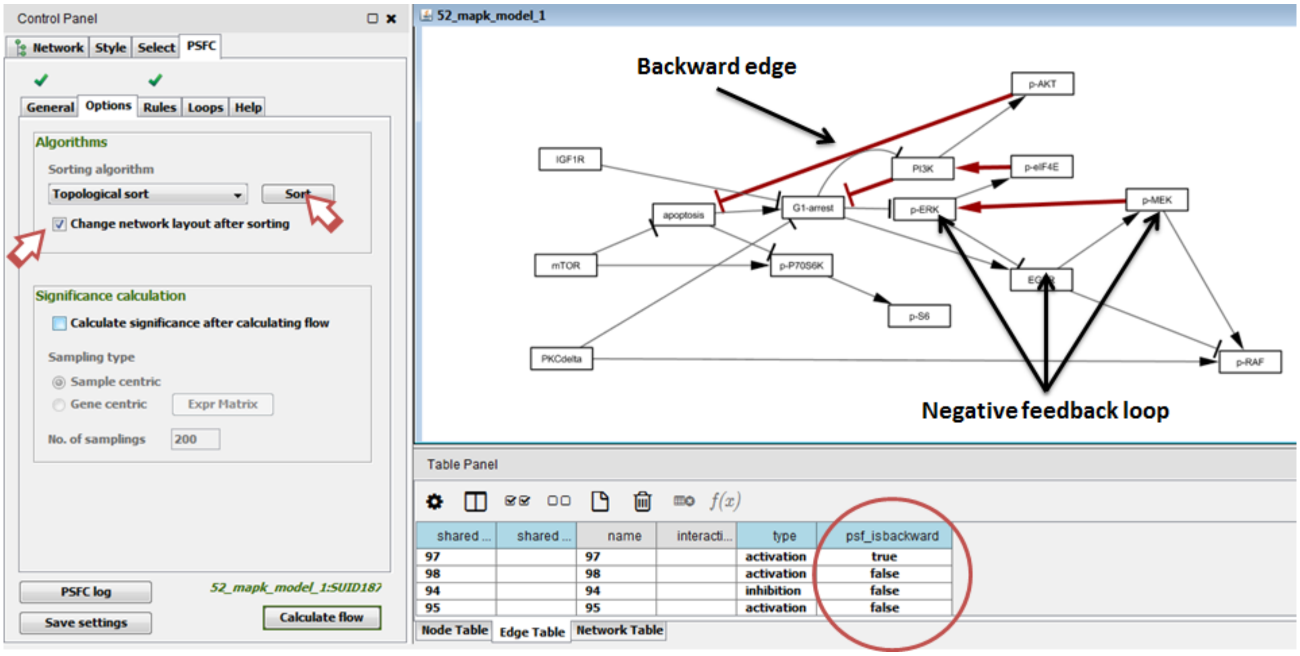

The backward edges introduce cycles in the graph, complicate signal flow propagation on the pathway. Among the three choices for loop handling presented in the **Loops** tab, we will choose the **Iterate until convergence option,** with its parameters: **Convergence threshold** = 1%, **Maximum number of iterations** = 50. This means that the flow propagation will be performed on the whole pathway for multiple times, updating the signal flow on the nodes after each iteration, until reaching convergence. A convergence is achieved, if the relative signal change between two consecutive iterations is less than **1%** for all the nodes in the pathway. If convergence is not achieved, the algorithm will stop after **50** iterations.

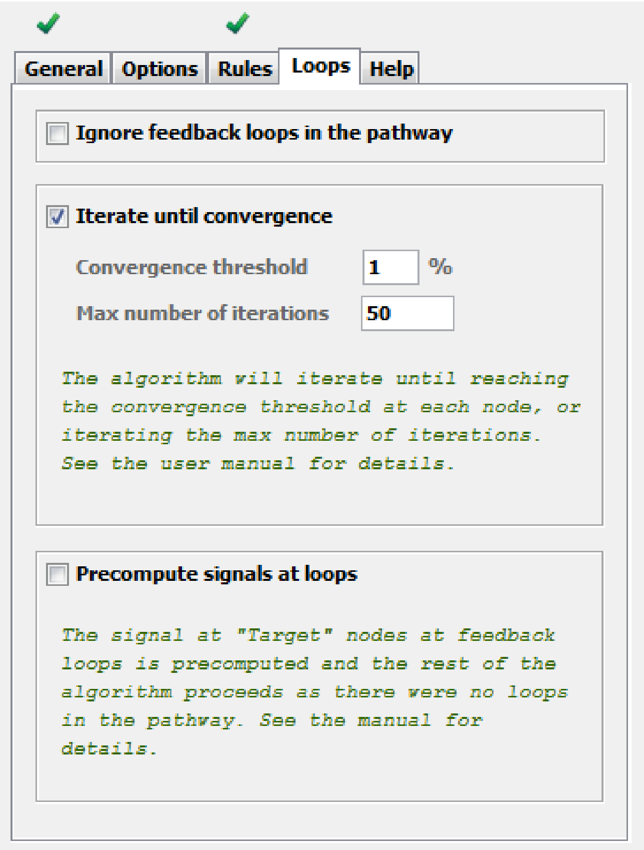

5. Calculate flow

Now that the rules are set, we can calculate the flow on the pathway. Before that, recall that all the nodes had **fc** values of 1. This means that all the nodes have the same value as the control state, thus no signal will

be propagated in the network (you may check this by calculating the flow). Thus, we will change the **fc** value of the IGF1R node to 0.1, down-regulating it, and will calculate the signal propagation resulting from this perturbation.

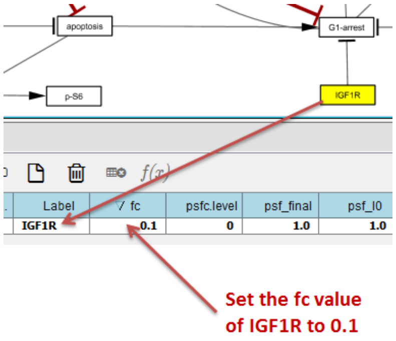

The flow is calculated by clicking the **Calculate flow** button at the bottom of the **PSFC** panel.

Note that a number of columns will appear in the node and edge attribute tables, that describe the PSF values of nodes and edges during signal propagation on the network: from lower levels (input nodes) to higher levels (sink nodes). There is a column named **psf_final**, which keeps the final output of PSFC.

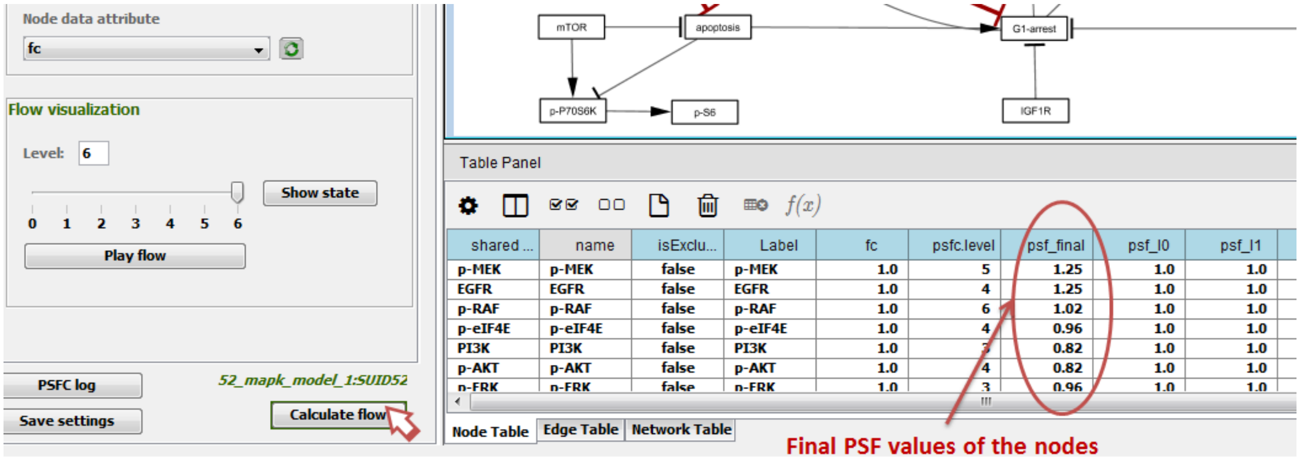

6. PSFC output

Besides the signal values in the node and edge attribute tables, PSFC tracks a log file, where the input, the option and the results of the computation are stored. The log file may be accessed by clicking the **PSFC log** button at the bottom of the **PSFC** panel. According to the results of our calculations, convergence was reached at iteration 7. The psf signal results can be found in the MAPK_configurations_v2.rar, the file MAPK_scores_output.xlsx.

```
Iteration: 5

Iteration: 6
Reached convergance at iteration: 7

Success: psf computation complete!
PostProcessed graph:
Graph{
nodes={0=Node{ID=0,name=PKCdelta,value=1.0,level=0,signals=[1.0, 1.0, 1.0, 1.0, 1.0, 1.0, 1.0]
, 1=Node{ID=1,name=IGF1R,value=0.1,level=0,signals=[0.1, 0.1, 0.1, 0.1, 0.1, 0.1, 0.1]
, 2=Node{ID=2,name=p-S6,value=1.0,level=3,signals=[1.0, 0.97, 0.94, 0.95, 0.95, 0.95, 0.95]
, 3=Node{ID=3,name=p-P70S6K,value=1.0,level=2,signals=[1.0, 0.97, 0.94, 0.95, 0.95, 0.95, 0.95]
, 4=Node{ID=4,name=apoptosis,value=1.0,level=1,signals=[1.0, 1.07, 1.12, 1.1, 1.11, 1.1, 1.1]
, 5=Node{ID=5,name=mTOR,value=1.0,level=0,signals=[1.0, 1.0, 1.0, 1.0, 1.0, 1.0, 1.0]
, 6=Node{ID=6,name=G1-arrest,value=1.0,level=2,signals=[1.29, 1.36, 1.41, 1.4, 1.4, 1.39, 1.39]
, 7=Node{ID=7,name=p-ERK,value=1.0,level=3,signals=[0.87, 0.97, 0.95, 0.98, 0.96, 0.97, 0.96]
, 8=Node{ID=8,name=p-AKT,value=1.0,level=4,signals=[0.87, 0.79, 0.81, 0.81, 0.82, 0.82, 0.82]
, 9=Node{ID=9,name=PI3K,value=1.0,level=3,signals=[0.87, 0.79, 0.81, 0.81, 0.82, 0.82, 0.82]
, 10=Node{ID=10,name=p-eIF4E,value=1.0,level=4,signals=[0.87, 0.97, 0.95, 0.98, 0.96, 0.97, 0.96]
, 11=Node{ID=11,name=p-RAF,value=1.0,level=6,signals=[1.02, 1.01, 1.02, 1.02, 1.02, 1.02, 1.02]
, 12=Node{ID=12,name=EGFR,value=1.0,level=4,signals=[1.23, 1.22, 1.27, 1.24, 1.25, 1.24, 1.25]
, 13=Node{ID=13,name=p-MEK,value=1.0,level=5,signals=[1.23, 1.22, 1.27, 1.24, 1.25, 1.24, 1.25]
}
edges=[Edge{source: ID=0; name=PKCdelta; target: ID=11; name=p-RAF; type='activation',; weight='0.29',; rank='0',; loopCount='0'}
, Edge{source: ID=0; name=PKCdelta; target: ID=6; name=G1-arrest; type='inhibition',; weight='0.33',; rank='0',; loopCount='0'}
, Edge{source: ID=1; name=IGF1R; target: ID=6; name=G1-arrest; type='inhibition',; weight='0.03',; rank='0',; loopCount='0'}
, Edge{source: ID=3; name=p-P70S6K; target: ID=2; name=p-S6; type='activation',; weight='1.0',; rank='0',; loopCount='0'}
```

The signals of the apoptosis node after each iteration

Additionally, the sorting-level-wise PSF values of the nodes after the last iteration are stored in an .xls file, with the name of the network. The file is located in the Cytoscape directory, usually in the c:\Users\User\CytoscapeConfiguration\app-data\PSFC\ path.

7. Visualize the flow

The flow propagation on the last iteration is visualized based on level-wise PSF values of nodes. The signal is visualized from lower levels of the pathway to the highest ones. Note that the layout of the pathway does not correspond to the sorted order, as shown in step 4, as the option for changing the network layout was not chosen.

The flow propagation is visualized based on the signal passing from the inputs of the network to its outputs, from lower levels to higher. In case of multiple iterations, the last iteration flow is shown. To see the original node values, simply hit the "home" button among the flow control buttons, or slide to level 0. The final state of the signals on the pathway is at the last level, or time-step. To see the last state, hit the "end" control button, or slide to the very end of the level slider. The intermediate states are visualized with forward and reverse controls.

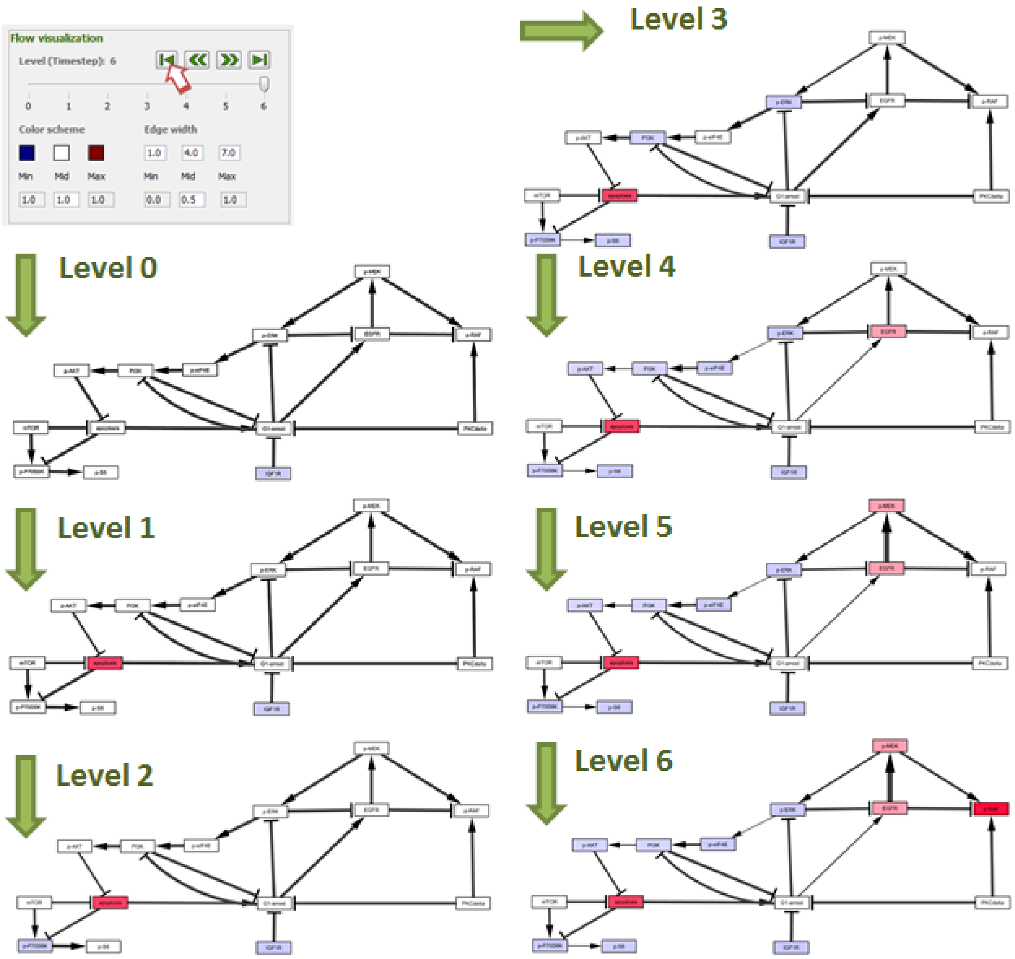

Note that the nodes are colored from blue to red, for signals from low to high. The median point is adjusted to be equal to 1, as it corresponds to the normal state. The middle signal for edges is also chosen to be 1, and the width of edges corresponds to the signal intensity. The colors, middle node signal value, edge width range and middle edge signal value are adjustable by the user. See the section *GUI components* for details.

GUI components

General tab

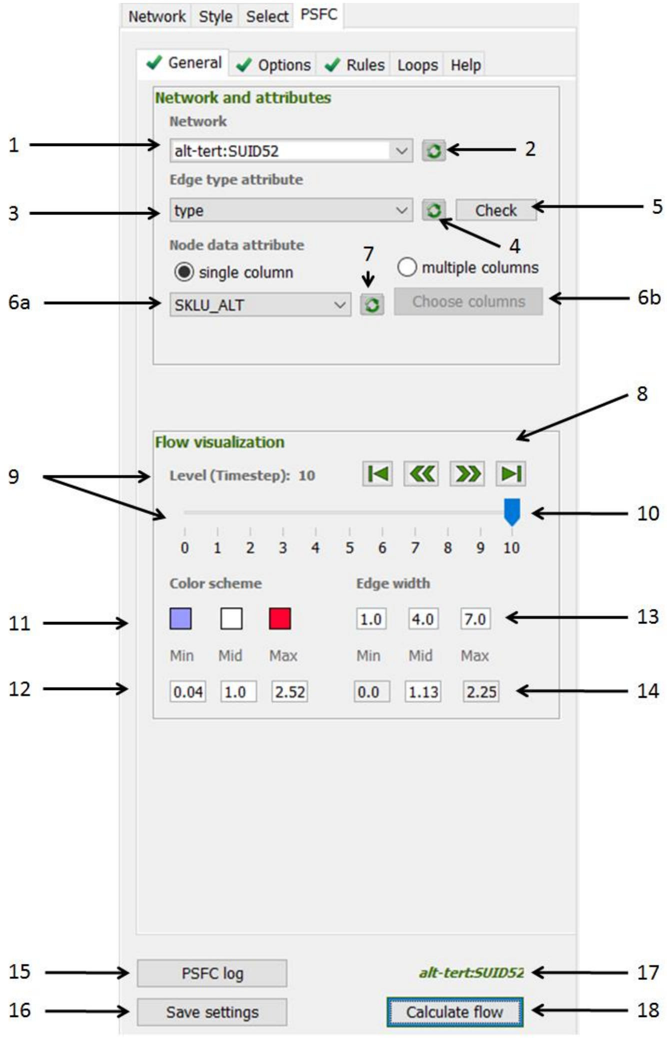

Contains the following components:

1. *Network* combo-box: for selection of the network under interest. The networks are represented in the form *networkName*:SUID*networkSUID*. If the chosen network is in the current view the network label (15) is green, otherwise it is red.
2. Refresh button for *Network* combo box - updates the list of currently loaded networks. Should be used after a new network has been loaded or removed.
3. *Select Edge type attribute* combo box - for selecting the Edge type attribute from the selected network's Default Edge Table. The Edge type attribute is used for defining the types of edges in the network and further setting edge type specific rules (see *Flow rule configurations* section).

4. Refresh button for *Select Edge type attribute* combo box - updates the list of columns of the selected network's Default Edge Table. Should be used when a column is added or removed from the table.
5. *Check* button for *Select Edge type attribute* combo box - shows the list of unique values in the selected Edge type column. The list is just for checking all the unique types of edges in the network, as well as for copying those for the rule configuration file (see *Flow rule configurations* section).
6. *a) Select Node data attribute* combo box - for selecting the Node data attribute from selected network's Default Node Table. The Node data attribute is used for setting the initial values of nodes for flow calculation. Node data values can be either row of logarithmic expression values, fold change or log-fold change of expression values, etc.

   b) ***New in 1.1.2!*** Select multiple columns for Node data attribute to compute perform PSFC calculations for more than one sample. Select the "multiple columns" radiobutton. Then choose the column names by holding the *Shift* key (consecutive columns) or the *Ctrl* key (non-consecutive columns) and press "Select", as shown in figure below. The PSF calculations will be performed with the specified options as in the case of a single column, but for several columns in a row. In addition to backup files created for each column, a 'summary.xls' file will be generated were the final psf and p values for each column will be presented. The files will be stored in the PSFC app directory.

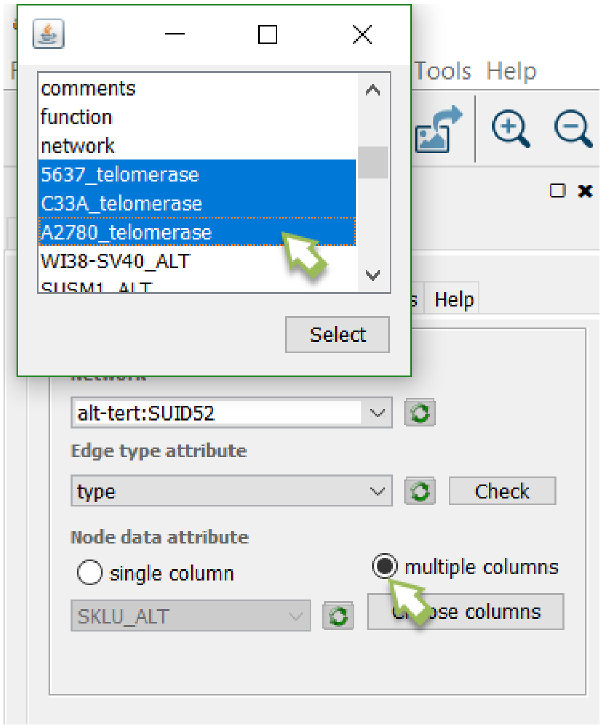

7. Refresh button for *Select Node data attribute* combo box - updates the list of columns of the selected network's Default Node Table. Should be used when a column is added or removed from the table.

8. *Flow visualization* panel - for performing visual mapping of node and edge signals after SPF calculation.
9. *Level* textbox and slider - for setting the PSF state at the specified level (timestep) in the sorted network. A level of the sorted network specifies the distance of nodes from the pathway input. The signal is propagated from the 0 level to the highest level in the pathway (see Network sorting for details).
10. *Control* buttons - performs color gradient visual mapping based on the node signals, and edge width mapping based on the edge signals, at the chosen level. There is the *home* button, which shows the signals at level 0, also corresponding to original values of nodes, and the *end* button*,* which shows the last level or final signals. The intermediate states can be viewed with the *forward and reverse* buttons, as well as the slider. Note that in case of many iterations, the signals of the last iteration only are shown.
11. *Color scheme* for node signals – the user may adjust the color key to be used in continuous mapping of node signals onto the network. The default is blue-white-red.
12. *Node signal* range – the user may adjust the “middle” (value of a node signal, below which the signals are considered as low and above which – as high), “min” and “max” values for node signals. By default, the middle signal is the average of the minimum and the maximum signals in the network. Note, that the middle value cannot be less than the minimum or more than the maximum.
13. *Edge width* range – the maximum and minimum edge widths corresponding to the highest and the lowest edge signals, for passthrough mapping to be applied on edges in the pathway. The middle width can be adjusted within the [min, max] range.
14. *Edge signal* range – the lowest, middle and the highest signals of edges in the network. The middle signal is the average between the lowest and the highest signals. It can be adjusted within this range by the user.

15. *PSFC log* button – Open the PSFC log file in the default text browser. If the file is not opening, open it manually, from the path indicated in the Cytoscape Task Manager.
16. *Save settings* button – saves the options from all the tabs.
17. *Network* label – shows currently chosen network.
18. *Calculate flow* button - Performs PSF calculation on the selected network.

Options tab

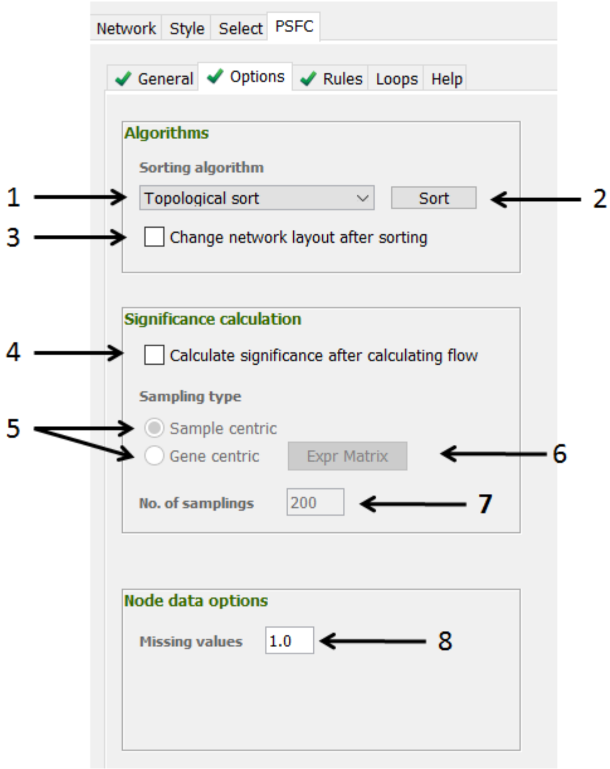

Contains the following components:

1. Sorting algorithm combo box - for selection of the sorting algorithm to be applied on the network. Currently only a modified version of topological sort algorithm is supported (see PSFC algorithms for details). In short, sorting assigns each node a level, which indicates the distance of that node from the nearest input node. The signal flow is propagated from the input nodes that have a level of 0 to the sink nodes that have highest level values.
2. *Sort* button - for applying selected sorting algorithm onto selected network and updating the network layout respectively.
3. *Change network layout after sorting* checkbox – if checked, the network layout will be changed by arranging the nodes from left to right – from lower to higher levels. Note that the original layout **will not be preserved.**
4. *Calculate Significance after calculating flow* checkbox – if checked, significance values of PSF values for all the nodes will be computed by Bootstrap resampling. The node input values are reshuffled during each resampling, and the PSF values are computed again. After multiple resamplings, the obtained distribution of PSF values for each node is used to calculate the z-score and the p value (see PSFC algorithms for details). The p values will appear in the "psfc_pval" node attribute column.
5. Radio buttons specifying the mode of Bootstrap resampling.

a. *Sample centric* – the values of the nodes in the network will be reshuffled among each other during resampling.

b. *Gene centric* – the value of each node is randomly chosen from a supplied distribution of node values. Such a distribution, e.g., may come from microarray chips, where the expression of a single gene is measured in several samples.

6. *Exp Matrix* button (for *Gene centric* option)– select the file, where the value distributions of nodes are specified. This is a tab delimited file, where the first column contains the node names, and the tab delimited values of the node are written in each row.

7. ***New in 1.1.2!*** *Missing value* text field – some of the nodes may have missing values in the dataset. In this case the user should set a default value. If the default PSFC rules are set, the nodes missing values should be assigned a value of 1.0, since this value will not affect the results of multiplication rules. If summation rules are set, then the missing values should be set 0. PSFC should be aware of the value assigned to the missing cases. This is important in case functions are applied onto nodes. PSFC will omit the missing values, and for this the user should specify which values are the “missing ones”.

Rules tab

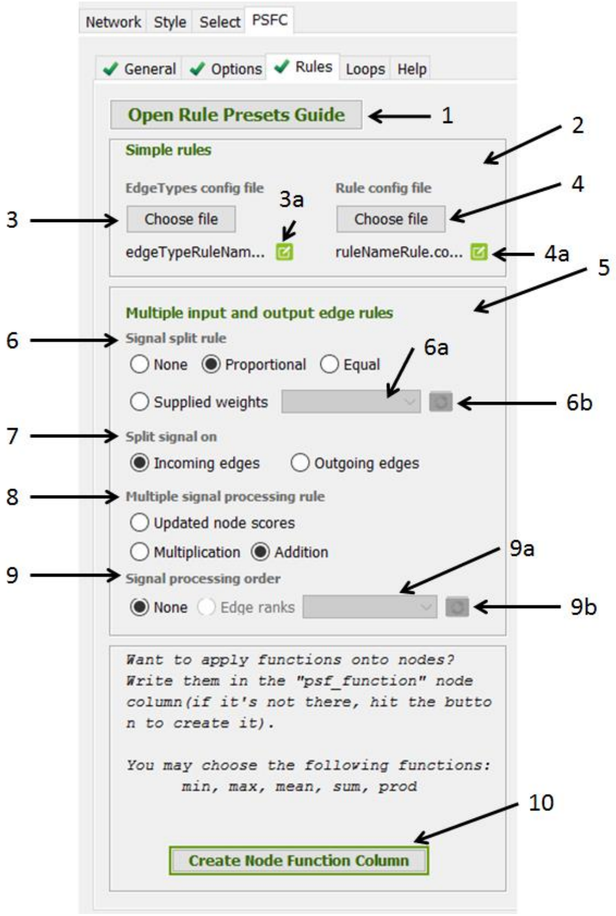

The *Rules* tab contains the following components:

1. *Open Rule Presets Guide* button – opens the PDF file, where preset options for the Rules tab are explained. If the PDF doesn't open for some reason, you should open manually from the PSFC app-data directory specified in the task monitor.
2. *Simple rules* panel - for setting edge type specific rules onto single edges. Defines how a signal is transferred from a source to a target node, via a single edge.
3. *Choose EdgeTypes config file* button - sets the configuration file containing edge type - rule name mapping. This should be a tab delimited file, where the first column contains edge types, and the second column contains the names of the functions assigned to each edge type.

   3a. The *EdgeTypes* config file may be easily accessed by hitting the "edit" icon: the system will attempt to open the file, or will show the file in its folder otherwise.
4. *Choose Rule config file* button - sets the configuration file containing function name - function mapping. This should be a tab delimited file, where the first column contains the function names, and the second column contains the function specifications corresponding to each name. The

functions should be of the form *f(source, target)*, where *source* is the source node signal, and *target* is the target node value.

4a. The *EdgeTypes* config file may be easily accessed by hitting the "edit" icon: the system will attempt to open the file, or will show the file in its folder otherwise.

5. *Multiple input and output edge rules* panel - for setting rules to process signals from multiple edges starting from a single source node or ending on a single target node.
6. *Signal split rule* - specifies whether to split multiple edges outgoing from a single source (or incoming to a single target node), (i) by dividing to the number of edges (*Equal*); (ii) by assigning weights to edges proportional to relative values of the target nodes (or relative signals of the source nodes) (*Proportional*); (iii) by assigning the edges user supplied weights (*Supplied Weights*); (iv) alternatively there can be no splitting at all (*None*).

   6a. The CyColumn in Default Edge table of the selected network, which contains edge weight attribute values; should be of Floating Point type; is active when *Supplied weights* option is selected.

   6b. Refresh button for supplied weights column -updates the list of columns of the selected network's Default Edge Table; should be used when a column is added or removed from the table.
7. *Split signal on* - specifies which edges to apply splitting on: *Incoming:* multiple edges ending on a single target node; *Outgoing* – multiple edges starting from a single source node.
8. *Multiple signal processing rule* - specifies how multiple signals received by a single node should be processed - be added (*Addition*) or multiplied (*Multiplication*) and replace the previous node signal, or the signals should be processed one after another, each of them replacing the previous node signal (*Updated node scores*).
9. *Signal processing order* – if the *Multiple signal processing rule* is set to *Update node scores,* the order in which the edges are processed matters. If set to *None,* the edges will be processed one after the other – in random order; alternatively the user may supply *Edge ranks*, defining the relative priorities of the edges: the smaller the rank, the sooner the edge will be processed.

   8a. The CyColumn in Default Edge table of the selected network, which contains edge rank attribute values; should be of Floating Point type; is active when *Edge ranks* option is selected.

   8b. Refresh button for edge ranks column - updates the list of columns of the selected network's Default Edge Table; should be used when a column is added or removed from the table.

10. *New in 1.1.2!* The user may also specify the rule for treating multiple incoming edges on specific nodes. The rule should be specified in the "psf_function" column. If no such column exists, hitting the "Create node function column" button will generate such a column in the Cytoscape node table. The function for each node may then be written in the respective row of this column.

The rules suggested are:

min: The minimum value of the incoming signals will be assigned to the node.

max: The maximum value of the incoming signals will be assigned to the node.

mean: The average signal among the incoming ones will be assigned to the node.

sum: The sum of all the incoming signals will be assigned to the node.

prod: The product of multiplication of all the incoming signals will be assigned to the node.

Note: the missing values are not taken into consideration while processing the node functions. This means the following. Let's say, there are three incoming signals with values 1.2; 3.2 and 1.0, and the missing value for a node (Options tab) is set to be 1.0. If the user specifies a 'min' function on this node, the minimum of 1.2 and 3.2, which is 1.2, will be set (the value of 1.0 will be considered as missing and will be omitted). If all of the incoming signals have missing values, the node will be assigned a signal equal to the missing value as well.

Loops tab

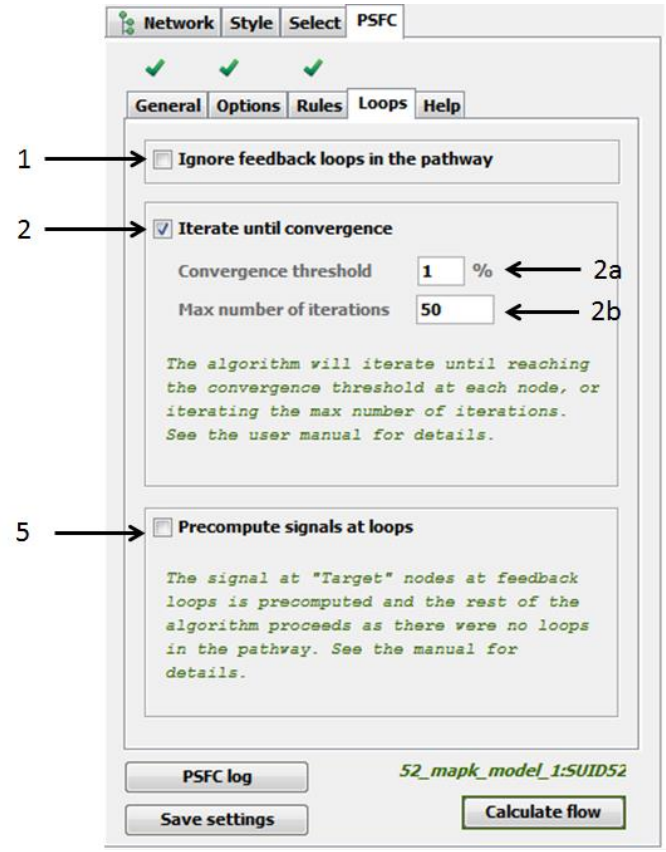

The *Loops* tab specifies how to handle feedback loops in a pathway. It has the following three options:

1. *Ignore feedback loops in the pathway* checkbox – if checked, the backward edges that form feedback loops will not be considered during PSF calculations.
2. *Iterate until convergence* checkbox – if checked, the PSF calculations will consider loop forming backward edges as other edges in the pathway, and the algorithm will run for several rounds until reaching convergence. Convergence in reached if the relative percent difference between node signals in two consecutive iterations is less than the specified threshold (*2a*). If convergence is not reached, the computation will finish after the specified maximum number of iterations (*2b*).
3. *Precompute signals at loops* checkbox – if checked, the signal at backward edges is firstly computed, and their target node values are updated. Afterwards, the PSF calculation is run on the whole network in *Ignore feedback loops* mode.

PSFC algorithms

Network sorting

For computation of pathway signal flow propagation, the network should initially be sorted based on distances of intermediate nodes from the input nodes, where input nodes, i.e. nodes that don't have incoming edges, should be assigned level 0. In order to see network sorting results before pathway flow calculation, the user may press the Sort button (4) in Network tab panel. PSFC will sort the selected network and optionally apply the level based layout onto network view. psfc.level column will appear in the default node table of the selected network, where the level of each node will be indicated.

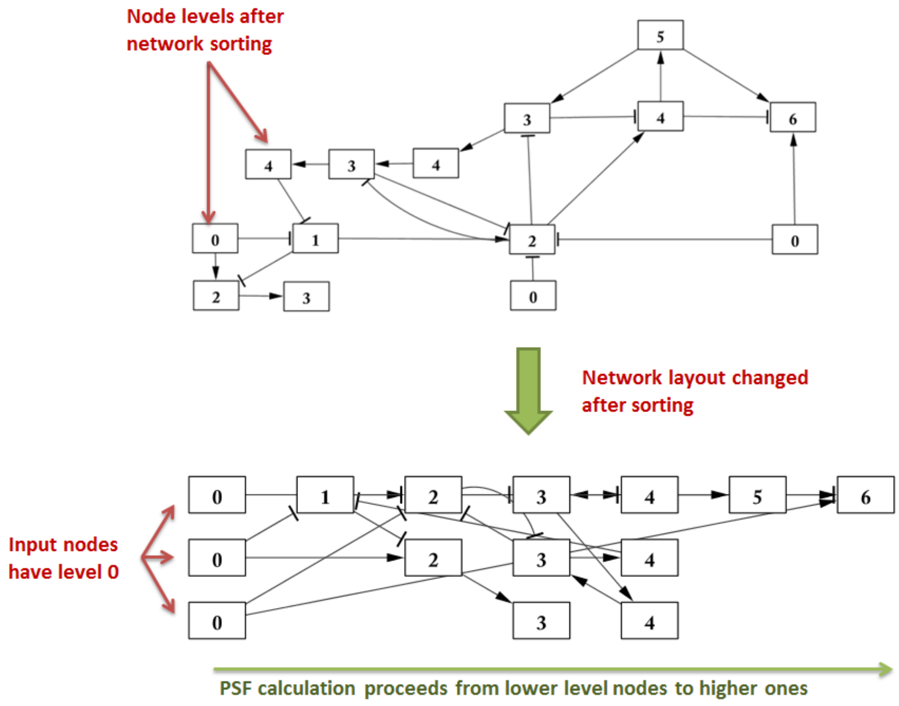

Currently, only a modified version topological sort algorithm is provided as an option. For directed acyclic graphs (DAGs) topological sorting algorithm orders the vertices of a graph in a way that for every edge uv for nodes u and v, u comes before v in the ordering. The algorithm uses JGraphT's TopologicalOrderIterator, but considers the fact that the graph may have more than one input vertex. Biological pathways, however, mostly contain cycles that represent positive or negative feedback loops. For sorting cycle-containing graphs, we first remove backward edges, which are found with a modified depth-first search algorithm (which also accounts for multiple input nodes). After removing the backward edges, the resulting graph is sorted with topological sorting algorithm, and finally the backward edges are restored in the graph.

Flow propagation rules

In biological signaling networks, functional interaction types can be broadly defined as activation or inhibition, while the range of physical and regulatory interactions is much wider (phosphorylation, binding, dimerization, ubiquitination, etc). Because of different types of interactions, each edge in a pathway should be associated with a different function that will describe how signal will propagate from its source node to the target.

Rules for signal flow propagation for single edges are defined with two files.

- *EdgeTypes config* file should be a tab delimited file, where the first column indicates the edge types, and the second column indicates rule names corresponding to each edge type. All the edge types in the selected network should be present in the config file in order for PSF calculation to be performed.
- *Rule config* file should be a tab delimited file, where the first column indicates the rule names, and the second column indicates the functions corresponding to each rule name. All rule names corresponding to all the edge types in the selected network should be present in this file. Functions are defined as mathematical equations of the form *f*(source, target), with variables named “source” and “target” for source and target nodes respectively. The user defined edge-type functions are parsed with Exp4j Java library [] for symbolic operations. The parsable functions include a number of mathematical operations, including *sin*, *cos, log* (natural logarithm), and constants, such as *Math.PI, Math.E.*

Rules for multiple incoming and outgoing signals are applied for the cases when multiple edges go out of a single source node (multiple outgoing edges), or multiple edges go to a single target node (multiple incoming edges).

Signal splitting: Generally, intensity of interactions between molecules largely depends on their concentration and activation state. However, if one type of molecule has several interacting partners, those may compete with each other, and the interaction capacity of the molecule may be “split” between those partners. Following this idea, PSFC is implemented with different options for splitting the signal among multiple outgoing or incoming edges.

The splitting of the signals is performed as follows:

(i) Edges are assigned weights according to the splitting rule. If the rule is set to “Equal”, the weight of each edge is calculated by dividing 1 to the number of edges. If the rule is set to “Proportional”, and edges are split on edges outgoing from a single source node, each edge gets a weight equal to the relative signals of its target nodes. If edges ending at a single target are split with the “Proportional” rule, the relative source signals are used for weight assignment. Alternatively, the user may supply weights of their choice.

(ii) The assigned edge weights are multiplied by the source node signal, if splitting is performed on multiple outgoing edges, or by the target node value, in case of splitting on multiple incoming edges;

(iii) Finally, the edge-associated function is applied to the weighted source and target node signals.

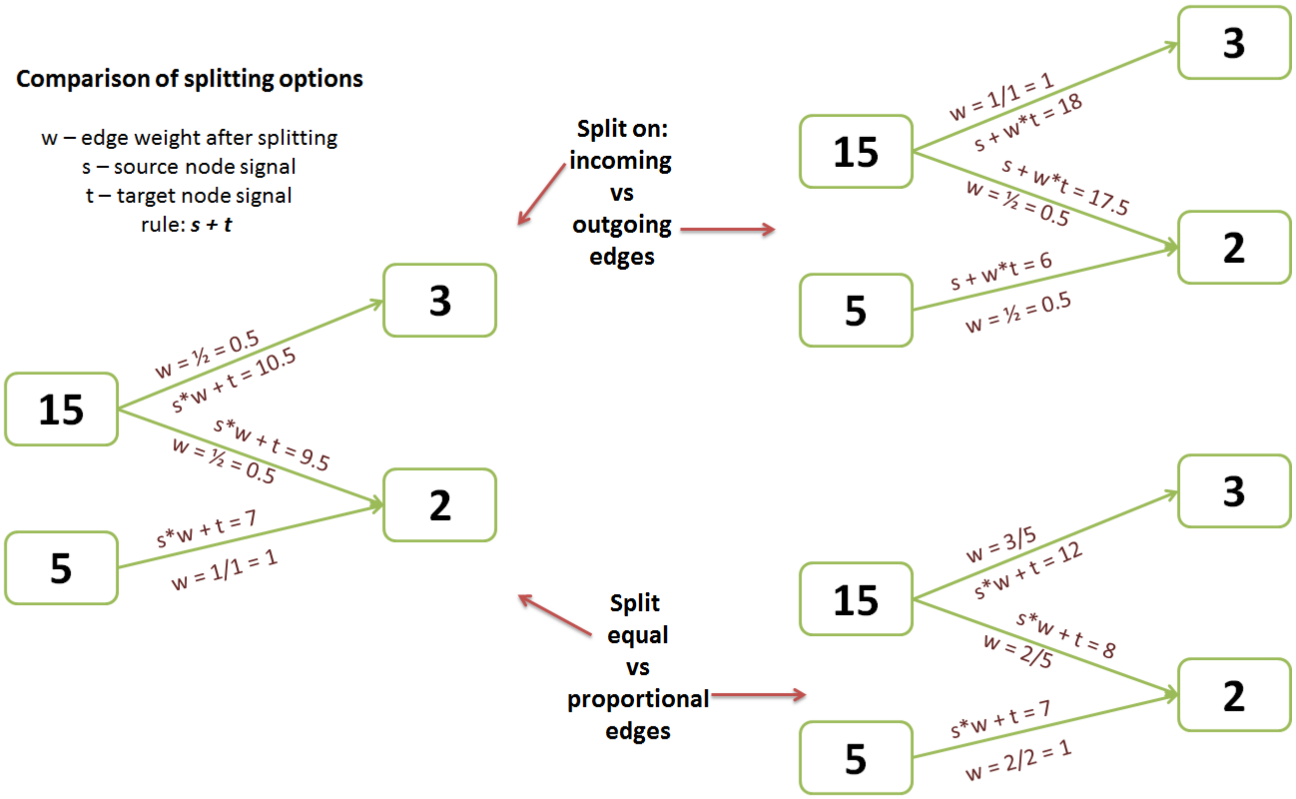

Multiple signal processing: Multiple signals entering a single target node may be processed in one the following three ways: those may be processed separately and the resulting signals added to each other (i) or multiplied (ii), or those may be processed in sequence, by updating the signal at a target node, each time an edge is processed (iii). The order, in which the edges are processed in the last case may be adjusted by the user by defining rank-based edge order (see the User Manual for details).

New in 1.1.2! **Node functions**: In addition to the multiple signal processing rules mentioned above, specific nodes in the network may be chosen to be treated differently. Five types of rules are currently implemented: *min, max, mean, sum* and *prod.* The *min, max and mean* functions take the minimum of all the non-missing signals. The *sum* and *prod* functions take the sum and product of the non-missing values respectively. Missing values (as set by the user in the node *Missing value* text field of the Options tab) are omitted from the calculations. If all the incoming signals are equal to the missing value, the target node is also assigned the missing value.

Pathway flow calculation

Pathway flow calculation is the process of signal propagation from nodes at the first level to the nodes at the last level of the network. At each level assigns weights to the edges, according to the options set with *Signal split rule* and *Split signal on* (button group no. 5 and 6 in the *Rules* tab). For each edge, the signal at the edge is computed based on the edge type-specific rule and the source and target node values, and multiplied by its weight. If a node has several incoming edges, the edge signals are processed and accumulated in the target node, according to *Multiple signal processing rule* and *Signal processing order* (button group no. 7 and 8 in the *Rules* tab).

To perform pathway flow calculation on the selected network, after setting the respective options simply press the *Calculate flow* button. Nodes and edge signals will be updated and the signals of all the nodes and edges will be visible in newly created or updated columns in default node and edge tables of the selected network in Cytoscape. The column names will have the suffix "psf_l" followed by the level value. Signal values are also kept in the score backup xls file, kept in the apps directory, usually located at C:\Users\User\CytoscapeConfiguration\app-data\PSFC\ (the address is indicated in TaskMonitor). The log of PSF calculation steps are debugged in PSFC log file, which is located in the apps directory, usually located at C:\Users\User\CytoscapeConfiguration\app-data\PSFC\PSFC.log, and can be opened by pressing the *PSFC log* button in the bottom of the panel.

Flow visualization

The *Flow visualization* panel allows the user to see the updated node signals by applying color based visual mapping of the values in the "psf _l" columns. The *Contorl* buttons allow for visualizing the network at different levels, or time-steps, which will show the node signals at the state when the flow propagation has reached the nodes of the level indicated on the levels' slider or level textbox. In case of iterative loop handling, the pathway flow is visualized for the last iteration only.

To see the original node values are visualized at level 0, which is reached by the "home" button among the flow control buttons, or with the slider. The final state of the signals on the pathway is at the last level, or

time-step. To see the last state, hit the “end” control button, or slide to the very end of the level slider. The intermediate states are visualized with forward and reverse controls.

Note that the nodes are colored from blue to red, for signals from low to high. The median point is adjusted to be equal to 1, as it corresponds to the normal state. The middle signal for edges is also chosen to be 1, and the width of edges corresponds to the signal intensity. The colors, middle node signal value, edge width range and middle edge signal value are adjustable by the user. See the section GUI components for details.

PSFC

Pathway Signal Flow Calculator

© 2017, Lilit Nersisyan, IMB NAS RA, Arsen Arakelyan, IMB NAS RA, Graham Johnson, UCSF, Megan Riel-Mehan, UCSF, Alexander Pico, Gladstone Institutes.

Licensed under: GNU General Public License version 3.

Discussion group https://groups.google.com/forum/#!forum/psfc-discussion-group

Correspondence:

Lilit Nersisyan,

l_nersisyan@mb.sci.am

Group of Bioinformatics,

Institute of Molecular Biology NAS RA

7 Hasratyan str. 0014, Yerevan, Armenia

March, 2017
