# Supplementary material for: PSFC: a Pathway Signal Flow Calculator App for Cytoscape
Source: F1000Res. 2017 Apr 3;4:480. Originally published 2015 Aug 5. [Version 2] doi: 10.12688/f1000research.6706.2 (PMC4706054; doi:10.12688/f1000research.6706.2)
Supplement: Peer review history

# Peer review: Referee response for version 1

**DOI:** 10.5256/f1000research.7203.r10923

**Published:** 2015-11-27

**Authors:** Maarten Altelaar, Linsey Raaijmakers

**Affiliations:** 1 Bijvoet Center for Biomolecular Research, Utrecht University, Utrecht, Netherlands; 2 Biomolecular Mass Spectrometry and Proteomics, Utrecht University, Utrecht, Netherlands

The authors present PSFC, a cytoscape app for the analysis of pathway signal flow to indicate pathway activity states. A user manual is presented with some example datasets.

In the manual the various steps are very well described.

My assessment for this paper is 'Approved', the app helps in visualization pathway flow and generates an easy interpretable network.

Just some minor comments and recommendations:

 

- The flow visualization part and the Rules tab in the control panel is not completely visible.  The bottom part falls outside the panel.  And also the 'Min', 'Mid', 'Max' text boxes are very small, making it difficult to modify the numbers inside these boxes.
- When pathways are loaded from ReactomeFIPlugin, the edge annotation column contains often more than 1 entry. This causes problems in the flow calculation steps. An idea is to split this column into the separate entries and check which entry is described in the edgeType config file.
- When the pathway is loaded from a plugin inside Cytoscape, like Reactome, the network file is not saved yet. This caused problems with writing of the results output file. It would be better if the network file could be automatically saved at this step.
- Some interactions could represent a complex, instead of an 'inhibition' or 'activation' signal, in this case there is not really a rule that would describe this situation, besides maybe source == target, but unfortunately I get an error when I use the '=' sign to describe an edge rule.

We have read this submission. We believe that we have an appropriate level of expertise to confirm that it is of an acceptable scientific standard.

# Response

**Published:** 2017-03-29

**Authors:** Lilit Nersisyan

**Affiliations:** Institute of Molecular Biology NAS RA, Armenia

**Response to the reviewers**

 We would like to thank the reviewer for their comments and recommendations. We have addressed those in the version 2 of the manuscript and in version 1.1.2 of PSFC.

 Here, the paragraphs containing responses to the reviewer’s comments are indicated with “Response:” in the beginning.

  

- The flow visualization part and the Rules tab in the control panel is not completely visible.  The bottom part falls outside the panel.  And also the 'Min', 'Mid', 'Max' text boxes are very small, making it difficult to modify the numbers inside these boxes.

 

**Response: **We have changed the layout of PSFC 1.1.2 accordingly. It is still possible that the user experiences distortions depending on the screen resolution. We would like to receive comments from users in such cases to make appropriate changes in further versions of the app.

- When pathways are loaded from ReactomeFIPlugin, the edge annotation column contains often more than 1 entry. This causes problems in the flow calculation steps. An idea is to split this column into the separate entries and check which entry is described in the edgeType config file.

**Response: **We understand the concern brought by the reviewers. However, while this feature would be useful in the particular case when pathways are loaded from the ReactomeFIPlugin, in other cases this would cause unnecessary complications. Therefore, we have left this split operation to the user: they may create an additional column containing the proper edge annotation entries.  

  

- When the pathway is loaded from a plugin inside Cytoscape, like Reactome, the network file is not saved yet. This caused problems with writing of the results output file. It would be better if the network file could be automatically saved at this step.

**Response:**  In PSFC the user should specify which network to perform calculations on. There may be many networks loaded into the Cytoscape session, while the user would desire to perform PSF on only one network. Therefore, we find that automatically saving the networks should not be a feature of PSFC. In case of issues, we advise the user to send us the details of the errors to perform appropriate fixes.

  

- Some interactions could represent a complex, instead of an 'inhibition' or 'activation' signal, in this case there is not really a rule that would describe this situation, besides maybe source == target, but unfortunately I get an error when I use the '=' sign to describe an edge rule.

**Response: **We thank the reviewers for this comment. We understand that the case of “source == target” can be handled by simply putting “source” in the edge rule. This will lead to source signals to be transferred to the targets as is. The cases of complex nodes are handled with the new feature of PSFC 1.1.2 – operator nodes. In addition to the rules assigned to edges, each specific node can also be assigned a function. E.g. if this node is a complex node it can take on the ‘min’ function, which means that the minimum signal of all the incoming nodes will be assigned to the target. Functions ‘min’, ‘max’, ‘mean’ and ‘prod’ are supported. The details are available in the user manual.

# Peer review: Referee response for version 1

**DOI:** 10.5256/f1000research.7203.r9853

**Published:** 2015-08-20

**Authors:** David Ruckerbauer

**Affiliations:** 1 Department of Biotechnology, University of Natural Resources and Applied Life Sciences, Vienna, Austria

The authors introduce an app for Cytoscape called 'Pathway Signal Flow Calculator' (PSFC). PSFC allows to simulate and visualize the propagation of signals through a pathway, considering both the pathway topology and input data for 'node activity', such as protein phosphorylation. The aim is to provide a flexible environment that supports the integration of various custom algorithms and data sets.

The paper describes several rules and algorithms for signal flow and demonstrates their effect on two small toy models as well as a bigger use case (MAPK signaling pathway), all in context of using PSFC.

Furthermore, the authors provide the use case with the corresponding input files as supplementary material, a very detailed step-by-step manual for the different options of PSFC and the source code of the app.

I have tested the app under Linux Mint 17.2 (64-bit), Mac OS X 10.7.5 (64-bit) and Windows Vista (32-bit) (see minor issues below).

While my assessment of this paper is 'Approved', I have come across some issues that are detailed below. Additionally to major and minor issues I have added a section with suggestions that I personally feel might improve PSFC. I would like to stress, however, that PSFC has full usability without considering any of the points in the latter section.

**Major Issues**

Figure 2: The definition of inhibition as 'source – target' seems counterintuitive, as a strong signal from the source should decrease the result in the target node. Should it be 'target – source'? This definition is also used by Isik *et al.* (Reference 4 in the paper) as well as in the document 'rules_presets.pdf' provided by the authors. Please be aware that both the text and the figures would need to be updated.

Figure 2 (F,G,H): 'Multiple input fields' is set to 'Update', the figures, however, correspond to 'Add'.

If it should indeed be 'Update' the values for the bottom right box are wrong in all three figures.

While all necessary input files for the use case are provided in the supplementary material the output is missing (apart from the screenshot of a log-file in the manual, which is not easy to read). This information should definitely be provided for all perturbations in order to be able to compare numerical values; a qualitative comparison is not enough, especially since all results are the same (up-regulation).

Ideally the toy model from Figure 2 would also be provided as an .xgmml-file, as this would enable readers to test and verify different settings.

**Minor Issues**

Clicking the link for reference 5 gives an error (page not found).

It is possible to install PSFC in Cytoscape running on Windows Vista 32-bit (with current versions of Cytoscape and Java), but running the calculation gives an error. I am aware that Cytoscape generally recommends using a 64-bit system, but if PSFC is for 64-bit systems only then this information could be added to the manual.

Redoing a calculation shuffles the sorting of the columns in the node / edge attribute table (in terms of the order of psf_l0, psf_l1, etc.), in Linux Mint.

Setting mTOR to an fc of 2 in the MAPK-example will lead to a final state where the box 'apoptosis' is completely white, even though the value is 0.75, and the box for 'p-RAF' is completely black (with a value of 4.59). This may or may not be an issue with the Cytoscape installation under Linux Mint.

I don't understand the purpose of the 'level textbox' in the flow visualization window. Does it only show the current maximum level?

Page numbers should be added to the manual.

**Suggestions**

Using a two color scheme (or maybe an easily customizable one) would improve the visualization. Right now even negative values are shown in the same color as positive ones and they are indistinguishable from low positive numbers.

An option to visualize the values for nodes and edges might be helpful.

Clicking the 'Play flow' button in the flow visualization window causes a pop-up which covers part of the network visualization. Maybe this pop-up could be forced to a bottom corner of Cytoscape.

The 'play flow' feature is on a very high speed if there is only a small number of levels. Ideally either the speed should be easily adjustable (and an endless mode available) or maybe the 'Show state' button could be removed and the visualization updated directly via the slider. This would allow to control the speed and direction of the visualization very easily. It would also solve the above mentioned issue of the pop-up.

Is the use of two config files (Edge Types config file and Rule config file) a requirement of Cytoscape or the parser? If not it would seem simpler to merge them and remove the redundant 'Function names'.

The file 'rules_presets.pdf' could be added to the manual instead of being a separate file, especially since there already is an extra pdf for the use case.

Additional toy models with the relevant input/output in order to test and verify 'significance calculation', handling feedback loops and edge weights might be helpful.

The paper explicitly mentions 'bench biologists' as potential users of PSFC. I feel that the app is described sufficiently in order to allow a user unfamiliar with modeling and/or Cytoscape to use it successfully. The bottleneck would probably be generating the necessary input model. Maybe a short paragraph in the manual pointing to the relevant sections in the Cytoscape manual as well as describing the creation and changing of node and edge attribute tables would remove this obstacle.

I have read this submission. I believe that I have an appropriate level of expertise to confirm that it is of an acceptable scientific standard.

# Response

**Published:** 2017-03-29

**Authors:** Lilit Nersisyan

**Affiliations:** Institute of Molecular Biology NAS RA, Armenia

**Response to the reviewer**

 We would like to thank the reviewer for their thorough revision of the manuscript, and for the useful suggestions. We have addressed the issues and suggestions and have incorporated them in the version 2 of the manuscript and in version 1.1.2 of PSFC.

 Here, the paragraphs containing responses to the reviewer’s comments are indicated with “Response:” in the beginning.

**Major Issues**

- Figure 2: The definition of inhibition as 'source – target' seems counterintuitive, as a strong signal from the source should decrease the result in the target node. Should it be 'target – source'? This definition is also used by Isik *et al.* (Reference 4 in the paper) as well as in the document 'rules_presets.pdf' provided by the authors. Please be aware that both the text and the figures would need to be updated.

 

**Response: **We agree with the reviewer. Although the user may set any rule they desire, “target – source” would be more intuitive for inhibition type of edges. We have updated the example and the figures respectively.

  

- Figure 2 (F,G,H): 'Multiple input fields' is set to 'Update', the figures, however, correspond to 'Add'.
  If it should indeed be 'Update' the values for the bottom right box are wrong in all three figures.

 

**Response: **We agree with the reviewer. The rule for multiple inputs is addition, while its mentioned as “update” in the figures 2 F,G,H. We have made respective changes.

  

- While all necessary input files for the use case are provided in the supplementary material the output is missing (apart from the screenshot of a log-file in the manual, which is not easy to read). This information should definitely be provided for all perturbations in order to be able to compare numerical values; a qualitative comparison is not enough, especially since all results are the same (up-regulation).

 

**Response: **We have presented the PSF scores of all the experiments in a separate document in the supplementary material.

  

- Ideally the toy model from Figure 2 would also be provided as an .xgmml-file, as this would enable readers to test and verify different settings.

 

**Response: **The example network in Figure2 is provided as an xgmml file. In addition we have provided instructions to replicate the settings applied in different subfigures.

**Minor Issues**

 Clicking the link for reference 5 gives an error (page not found).

 Update the link with https://www.iconceptpress.com/books/genomics-ii--bacteria-viruses-and-metabolic-pathways/11000061/1205000491.pdf.

 It is possible to install PSFC in Cytoscape running on Windows Vista 32-bit (with current versions of Cytoscape and Java), but running the calculation gives an error. I am aware that Cytoscape generally recommends using a 64-bit system, but if PSFC is for 64-bit systems only then this information could be added to the manual.

**Response: **This was explicitly mentioned in the Manual.

 Redoing a calculation shuffles the sorting of the columns in the node / edge attribute table (in terms of the order of psf_l0, psf_l1, etc.), in Linux Mint.

**Response: **Unfortunately, currently it is impossible to order the columns in Cytoscape. Currently, this as an ogoing task and will soon be addressed by the Cytoscape development team.

 Setting mTOR to an fc of 2 in the MAPK-example will lead to a final state where the box 'apoptosis' is completely white, even though the value is 0.75, and the box for 'p-RAF' is completely black (with a value of 4.59). This may or may not be an issue with the Cytoscape installation under Linux Mint.

**Response: **The versions 1.0.2 and 1.1.2 of PSFC allows for setting the color scheme based on custom preferences. So the user may get rid of complete white and black nodes.

 I don't understand the purpose of the 'level textbox' in the flow visualization window. Does it only show the current maximum level?

**Response: **The flow visualization is based on sequential mapping of the node and edge signals, from the input nodes (level 0), to the sink nodes (highest level).  The level textbox may thus be considered as an indication of time-step. In PSFC 1.0.2 and 1.1.2 we have made it non-editable and named it “time-step”, so that is more understandable to the user.

  Page numbers should be added to the manual.

**Response: **According to the reviewer’s comment, we have added page numbers to the manual.

**Suggestions**

 Using a two color scheme (or maybe an easily customizable one) would improve the visualization. Right now even negative values are shown in the same color as positive ones and they are indistinguishable from low positive numbers.

**Response: **Thank you for the suggestion. We have added a two-color scheme in PSFC 1.0.2, with option to specify also the middle color and signal value. Additionally, the user may also choose the range of edge widths.In PSFC 1.1.2 the the minimum and maximum values may also be modified by the user.  

 An option to visualize the values for nodes and edges might be helpful.

**Response: **The initial values of nodes and edges can be visualized by flow visualization at level of time-step 0. We have additionally described this in the manual.

 Clicking the 'Play flow' button in the flow visualization window causes a pop-up which covers part of the network visualization. Maybe this pop-up could be forced to a bottom corner of Cytoscape.

 The 'play flow' feature is on a very high speed if there is only a small number of levels. Ideally either the speed should be easily adjustable (and an endless mode available) or maybe the 'Show state' button could be removed and the visualization updated directly via the slider. This would allow to control the speed and direction of the visualization very easily. It would also solve the above mentioned issue of the pop-up.

**Response: **According to the reviewers suggestion, we have removed the “Play flow button” in PSFC 1.0.2 and added control buttons, so that the user is able to visualize the pathway states manually. This also removed the problem with the pop-up.

 Is the use of two config files (Edge Types config file and Rule config file) a requirement of Cytoscape or the parser? If not it would seem simpler to merge them and remove the redundant 'Function names'.

**Response: **The edge types config file describes the mapping between edge types and function names, while the rule config files assigns mathematical functions to each function name. The reason these two files are separate, is that multiple edge types can be treated with the same rule, and writing the same function for that rule multiple types in a file may produce errors. Thus, we think that keeping the mappings separate is more optimal. The instructions for using Figure 2 toy example of the manuscript, also describe the advantage of having two separate config files.

 The file 'rules_presets.pdf' could be added to the manual instead of being a separate file, especially since there already is an extra pdf for the use case.

**Response: **The “rules_preset.pdf” is to assist the user specifically in setting up the options for flow propagation rules. While the user may be familiar with the rest of the options in PSFC, this is intended to be a quick look-up guide for rules. Thus, we made it as a separate file, to free the user from scrolling through the manual every time they have a doubt which rule to use.

 Additional toy models with the relevant input/output in order to test and verify 'significance calculation', handling feedback loops and edge weights might be helpful.

**Response: **The toy network model for loop-testing has also been added in the supplementary material of the second version of the manuscript.

 The paper explicitly mentions 'bench biologists' as potential users of PSFC. I feel that the app is described sufficiently in order to allow a user unfamiliar with modeling and/or Cytoscape to use it successfully. The bottleneck would probably be generating the necessary input model. Maybe a short paragraph in the manual pointing to the relevant sections in the Cytoscape manual as well as describing the creation and changing of node and edge attribute tables would remove this obstacle.

**Response: **We agree with the reviewer. We have referred the user to respective pages of the Cytoscape manual, in the PSFC User Manual, where appropriate.
